# Serum receptor-interacting protein kinase 3 (RIPK3) is associated with transplant-free survival in acute liver failure

**DOI:** 10.1016/j.jhepr.2026.101962

**Published:** 2026-08-04

**Authors:** Gautam Mehta, Chengyi Ding, Ugo Soffientini, Jaime L. Speiser, Sarah Bahnassi, Lily Dara, Valerie Durkalski, William M. Lee, Constantine J. Karvellas, US Acute Liver Failure Study Group

**Affiliations:** 1 Institute for Liver and Digestive Health, University College London, London, UK; 2 Roger Williams Institute of Hepatology, Foundation for Liver Research, London, UK; 3 Department of Biostatistics and Data Science, Division of Public Health Sciences, Wake Forest University School of Medicine, Winston-Salem, NC, USA; 4 Department of Medicine, University of Southern California, Los Angeles, CA, USA; 5 Department of Public Health Sciences, Medical University of South Carolina, Charleston, SC, USA; 6 Division of Digestive and Liver Diseases, Department of Internal Medicine, University of Texas Southwestern Medical Center, Dallas, TX, USA; 7 Divisions of Hepatology and Critical Care Medicine, University of Alberta, Edmonton, Canada

**Keywords:** RIPK3, Acute liver failure, Multiorgan failure, Prognosis

## Abstract

**Background & Aims:**

Prognostication in acute liver failure (ALF) due to acetaminophen or drug-induced liver injury remains challenging. Biologically informed prognostic biomarkers may complement existing tools and improve early risk stratification. We aimed at investigating the associations between circulating mediators of inflammatory cell death and 21-day transplant-free survival (TFS) in ALF.

**Methods:**

We conducted a nested case-control study of 176 adults with ALF due to acetaminophen or drug-induced liver injury; they were selected from the Acute Liver Failure Study Group registry. Serum levels of receptor-interacting protein kinase 3 (RIPK3), mixed lineage kinase domain-like protein (MLKL), gasdermin D (GSDMD), and gasdermin E (GSDME) were measured at admission (Day 1) and Day 4. Associations with 21-day TFS were evaluated using multivariable logistic regression and receiver operating characteristic analyses. Machine learning methods were used to characterize patient heterogeneity and assess the robustness of biomarker-outcome associations.

**Results:**

At Day 21, 86 patients with ALF were alive without a liver transplant, whereas 90 died or received a liver transplant. Lower admission RIPK3 levels were independently associated with 21-day TFS after adjustment for clinically relevant covariates, including etiology, MELD score, and organ support requirements (adjusted odds ratio 1.10 per 1 ng/ml decrease; 95% CI 1.03–1.17). Incorporation of RIPK3 into clinical models at admission improved discrimination compared with established prognostic tools (AUROC 0.87; 95% CI, 0.81–0.92). The prognostic association of RIPK3 was most evident in patients with greater physiological severity at presentation.

**Conclusions:**

Serum RIPK3 is independently associated with TFS in ALF and improves the discriminative performance of clinical prognostic models. These findings support RIPK3 as a candidate prognostic biomarker in ALF.

**Impact and implications:**

In this study of 176 patients with ALF, lower serum receptor-interacting protein kinase 3 (RIPK3) levels were independently associated with 21-day transplant-free survival. The incorporation of RIPK3 into established prognostic tools improved discrimination between survivors and non-survivors. This study has particular clinical relevance to patients with ALF who had a higher burden of multiorgan failure, in whom the prognostic association of RIPK3 was more evident. Incorporating RIPK3 in current prognostic models may assist clinical decisions regarding which patients with ALF require transplantation *vs*. those who may recover with medical therapy; however, future prospective studies are needed to validate these findings.

## Highlights

• Prognostication in ALF remains challenging, with commonly used clinical scores demonstrating limited sensitivity.
• Lower serum levels of RIPK3 were independently associated with 21-day transplant-free survival in ALF.
• Incorporating RIPK3 into clinical models at admission improved discrimination of survivors and non-survivors.
• The prognostic association of RIPK3 was most evident in patients with greater physiological severity at presentation.

## Graphical abstract

**Figure:**
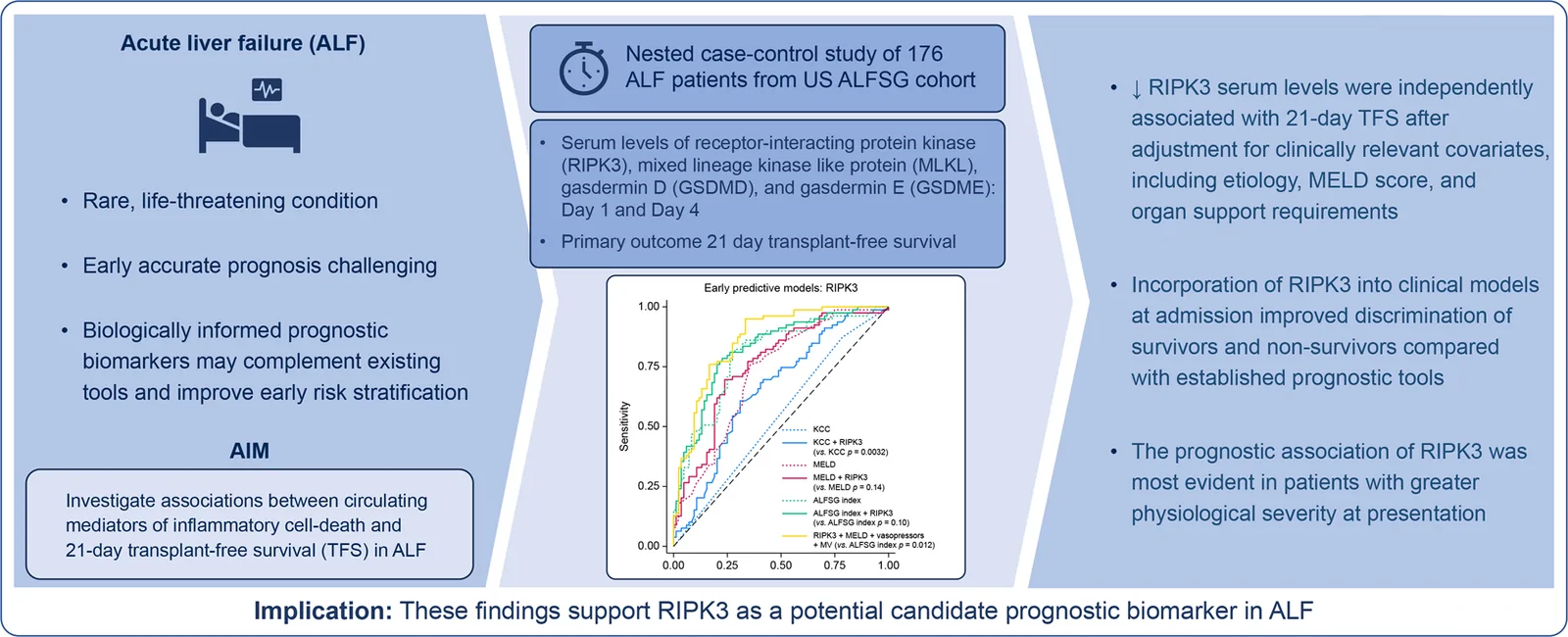

## Introduction

Acute liver failure (ALF) is defined by the occurrence of hepatic encephalopathy (HE) and synthetic dysfunction within 26 weeks of the first symptoms of liver disease.^1^ Currently, the most common cause of ALF in North America is acetaminophen (APAP).^2^^,^^3^ The most widely used prognostic model in patients with APAP-ALF is the King’s College Criteria (KCC).^4^^,^^5^ Although initially published in 1989, subsequent studies using KCC have shown relatively poor sensitivity of the APAP criteria, ranging 25–76%.^3^^,^[6], [7], [8], [9] More recently, the Acute Liver Failure Study Group (ALFSG) prognostic index also demonstrated limited sensitivity (41%) in APAP-ALF.^9^

Liver transplant (LT) in ALF presents substantial challenges because of the rapidity and severity of illness (*i.e*. high risk of cerebral edema and multiorgan failure), the potential for recovery without LT, and the presence of concomitant complex psychosocial issues (contraindications) in several patients.[10], [11], [12] Given the advances in critical care management of ALF, some patients may have a good outcome despite meeting KCC and potentially avoid unnecessary LT in the presence of improved prognostic markers or scores.^7^^,^^13^ There is an unmet need to discover and incorporate novel biomarkers of liver injury for prognostication of ALF and to determine which patients will require transplantation.

Inflammation and hepatocyte death underpin liver failure. Historically, necrosis and apoptosis have been considered the dominant modes of cell death in ALF;^14^ however, more recently, programmed forms of pro-inflammatory cell death have been described. Necroptosis is a form of programmed necrosis that has a similar pathway and mediators of activation as apoptosis (*i.e*., tumor necrosis factor receptor 1 [TNFR1], TNFR2, TNF-related apoptosis including ligand [TRAIL] 1-2, and Fas).^15^ However, under conditions of caspase-8 inhibition or ATP deficiency, this pathway leads to a lytic, pro-inflammatory cell death which morphologically resembles necrosis. The receptor-interacting protein kinases (RIPK) 1 and 3 are central to the necroptosis pathway through formation of the multimolecular necrosome complex, leading to phosphorylation of the effector protein mixed lineage kinase like protein (MLKL), which, in turn, forms pores within the cell membrane causing pro-inflammatory cell lysis. Importantly, necroptosis-independent and inflammation-dependent roles have been described for RIPK1, RIPK3, and MLKL.^16^ Although the contribution of necroptosis in pre-clinical models of APAP-induced liver injury has been controversial, the potential for RIPKs and MLKL to act as prognostic biomarkers of inflammation and cell death in ALF has not been studied in humans.[17], [18], [19], [20], [21], [22], [23]

Pyroptosis is a further, pro-inflammatory mode of cell death implicated in liver disease. In a similar fashion, pyroptosis is characterized by lytic cell death following activation and cleavage of the effector proteins, gasdermins. The best described pathway is through activation of caspase(s)-1/4/5, leading to cleavage of gasdermin D (GSDMD), which then oligomerizes and integrates within the plasma membrane.^24^ Gasdermin E (GSDME) is structurally similar, but it is cleaved by caspase-3, and therefore provides a link between apoptosis and pyroptosis.^25^ Both the forms of pyroptosis lead to lytic cell death and release of pro-inflammatory damage-associated molecular patterns (DAMPs). Again, pre-clinical studies support a causal role for GSDMD and GSDME in liver disease, GSDMD in ALF,[26], [27], [28], [29] and GSDME in steatohepatitis;^30^ however, their prognostic role in patients with ALF remains unknown.

In this study, we aimed at determining the association of necroptosis and pyroptosis pathway proteins (RIPK3, MLKL, GSDMD, and GSDME) with clinical outcomes in a well-characterized ALF patient cohort. We tested the hypotheses that (1) lower serum levels of these biomarkers are associated with 21-day survival in the absence of LT, and (2) the addition of these biomarkers improves the performance of currently used ALF prognostic models including KCC, model for end-stage liver disease (MELD), and the ALFSG index.

## Patients and methods

### Study design and participants

This was a case-control study nested within the ALFSG registry. The ALFSG registry consists of 2,631 adult patients with ALF admitted to one of the 32 enrolling clinical sites from 1998 to 2019. Eligible patients with ALF had both coagulopathy (international normalized ratio [INR] ≥1.5) and any grade of HE (as clinically defined by the classic West Haven criteria) within 26 weeks of the onset of symptoms, and had no evidence of chronic liver disease.^31^ All participating sites adhered to local institutional review board requirements, and written informed consent was obtained from the patient’s legal next of kin.

Consistent with ALFSG studies,^31^ the primary outcome of the current analysis was 21-day transplant-free survival (TFS). A total of 1,326 patients in the ALFSG registry were alive at Day 21 in the absence of LT, of whom 86 were randomly selected for the present analysis. Among the 1,303 patients who had non-TFS status, 90 were randomly selected (Fig. S1). Random selection was performed by personnel not involved in sample processing or statistical analysis for the paper, blinded to the clinical, biochemical, and demographic information of the patients other than the 21-day TFS status. A further eight healthy controls were analyzed for RIPK3, MLKL, GSDMD, and GSDME only.

Reporting of the analysis of this study followed the STROBE guidelines for reporting case-control studies.^32^

### Clinical variables

Demographic data (age, race, and sex) were collected alongside biochemical parameters, which were prospectively measured on Day 1 and Day 4 and included complete blood count, INR, transaminases, bilirubin, ammonia, creatinine, lactate, and phosphate. The need for organ support, such as mechanical ventilation (MV), vasopressors, and renal replacement therapy (RRT), was documented as a binary variable (yes/no) from Day 1 to Day 7.

Prognostic scores were calculated on Day 1 and Day 4. The KCC predicts poor outcome (death/LT) in APAP-ALF if: (1) pH <7.3, or (2) INR >6.5, creatinine >3.4 mg/dl, and high HE coma grade (grade 3 or 4). In non-APAP drug-induced liver injury (DILI), KCC is defined as: (1) INR >6.5, or (2) any three of the following criteria: age <10 or >40 years, duration of jaundice before HE >7 days, INR >3.5, and serum bilirubin >300 μmol/L.^4^ MELD is defined as:

10 × (0.957 × log[4] + 0.378 × log[bilirubin] + 1.12 × log[INR]) (1)

for dialysis patients and

10 × (0.957 × log[creatinine] + 0.378 × log[bilirubin] + 1.12∗log[INR]) (2)

for non-dialysis patients.^33^ The ALFSG index calculates the log odds (logit) for 21-day TFS as

2.67 - 0.95 × (HE) + 1.56 × (etiology) - 1.25 × (vasopressor) - 0.70 × ln(bilirubin) - 1.35 × ln(INR).^9^ (3)

HE = 1 if the coma grade is high (grade 3 or 4), etiology = 1 if APAP, and vasopressor use = 1 if present; otherwise, all are 0.

### Laboratory assays of RIPK3, MLKL, GSDMD, and GSDME

The following ELISAs were performed in duplicate according to manufacturer’s instructions: RIPK3 (Biorbyt, Durham, NC, USA; orb552189), MLKL (Biorbyt; orb443049), GSDMD (Abcam, Cambridge, UK; AB272463), and GSDME (Abbexa, Cambridge, UK; abx386859). Both Days 1 and 4 serum samples were analyzed in these assays.

### Statistical analysis

Differences between TFS and non-TFS status among patients with ALF were compared using the Chi-square test for categorical variables. Continuous variables were reported as medians with IQR and compared using the Wilcoxon rank-sum test. Logistic regression analysis was performed to examine the association of RIPK3, MLKL, GSDMD, and GSDME with 21-day TFS. Biomarker levels are reported as absolute values and, in sensitivity analyses, as multiples of the upper limit of normal ( × ULN) observed in healthy controls. Covariates considered were etiology, MELD, vasopressor use, need for RRT, and MV; variance inflation factor (VIF) indicated no significant multicollinearity (VIF ≤1.74 for all). Separate models were derived for biomarkers and covariates measured on Day 1 and Day 4, as well as for changes (Δ = Day 4 – Day 1) in biomarkers, using complete-case analyses. To explore patient heterogeneity, we applied factor analysis of mixed data (FAMD) to admission clinical variables for all ALFSG patients with ALF with available baseline data (n = 2,619), without incorporating biomarker levels or TFS outcomes. The resulting dimensions were used for k-means clustering to identify potential patient subgroups, and associations between biomarkers and 21-day TFS were subsequently evaluated within each cluster. Sensitivity analysis was performed using Gaussian mixture model (GMM) clustering to address uncertainty in cluster assignment.

Receiver operating characteristic (ROC) curve analysis was conducted to assess the significance of adding RIPK3, MLKL, and GSDME to existing prognostic scores (KCC, MELD, and ALFSG index). An additional model using Day 1 measurements of RIPK3 and covariates was derived by performing backward elimination with a *p* value threshold of 0.05. Comparisons of the area under the ROC curve (AUROC) statistics between models (*e.g*. KCC vs. KCC + RIPK3) were performed using the DeLong method.^34^ A 10-fold cross-validation was carried out to evaluate model generalizability, with AUROC calculated for each fold and averaged across all folds. The 95% CI for the mean AUROC was derived using the bootstrap method. R version 4.4.2 (R Foundation for Statistical Computing, Vienna, Austria) and SAS version 9.4 (SAS Institute, Cary, NC, USA) were used for the analysis. A two-sided *p* value of <0.05 was considered statistically significant.

## Results

### Patient characteristics

Of the 176 patients with ALF, 153 (87%) were diagnosed with APAP-ALF, whereas 23 (13%) developed ALF due to DILI. Furthermore, 86 patients spontaneously survived by Day 21 (TFS), 89 died, and one underwent LT. The most common causes of death were multiorgan failure (n = 43, 48%) and cerebral edema (n = 22, 25%); the cause of death was unknown in 24 (27%) patients.

Patient characteristics stratified by 21-day TFS status are shown in Table 1. There were no significant differences in age or sex between TFS and non-TFS. During the first 7 days of study admission, patients with TFS status required significantly less organ support than patients with non-TFS status, including MV (50% *vs*. 91%), vasopressor use (28% *vs*. 73%), and RRT (34% *vs*. 60%; *p* ≤0.0005 for all). They were also less likely to achieve grade 3 or 4 HE (54% *vs*. 89%) or to receive mannitol for intracranial hypertension (8% *vs*. 36%; *p* <0.0001 for both). Compared with patients with non-TFS status, patients with TFS status experienced fewer complications, such as seizures (0% *vs*. 17%), arrhythmias (16% *vs*. 37%), and gastrointestinal bleeding (5% *vs*. 19%; *p* ≤0.004 for all). On admission (Day 1), 12% of TFS patients and 26% of patients with non-TFS status met KCC (*p* = 0.018).

**Table 1:** Patient characteristics by 21-day TFS status.

	Spontaneous alive at Day 21 (TFS, n = 86)	Dead/LT at or before Day 21 (non-TFS, n = 90)	*p* values
Age (years)	39 (29–50)	41 (31–56)	0.222
Etiology			
APAP	75 (87.2)	78 (86.7)	0.915
DILI	11 (12.8)	12 (13.3)	
Male	21 (24.4)	29 (32.2)	0.251
Race			
White/Caucasian	74 (86.0)	66 (73.3)	0.090
African American	6 (7.0)	15 (16.7)	
Other	6 (7.0)	9 (10.0)	
Organ support (Days 1–7)			
Mechanical ventilation	43 (50.0)	82 (91.1)	<0.001
Vasopressors use	24 (27.9)	66 (73.3)	<0.001
Renal replacement therapy	29 (33.7)	54 (60.0)	<0.001
KCC (Day 1)	10 (11.6)	23 (25.6)	0.018
HE coma grade 3 or 4∗ (Days 1–7)	45 (53.6)	79 (88.8)	<0.001
ICP-directed therapies (Days 1–7)			
ICP monitor	2 (2.3)	22 (24.4)	<0.001
Mannitol	7 (8.1)	32 (35.6)	<0.001
Hypertonic saline	11 (12.8)	14 (15.6)	0.599
Barbiturates	6 (7.0)	17 (18.9)	0.019
Hypothermia	3 (3.5)	15 (16.7)	0.004
Sedatives	44 (51.2)	78 (86.7)	<0.001
Need for blood products (Days 1–7)			
Red blood cells	23 (26.7)	47 (52.2)	<0.001
Fresh frozen plasma	26 (30.2)	69 (76.7)	<0.001
Recombinant VIIA	0 (0.0)	5 (5.6)	0.027
Platelets	13 (15.1)	36 (40.0)	<0.001
ICU complications (Days 1–7)			
Seizures	0 (0.0)	15 (16.7)	<0.001
Arrhythmias	14 (16.3)	33 (36.7)	0.002
Gastrointestinal bleeding	4 (4.7)	17 (18.9)	0.004
Abnormal chest X-ray	40 (46.5)	25 (27.8)	0.010
Infection (Days 1–7)			
Positive urinary tract infection	22 (25.6)	17 (18.9)	0.285
Positive tracheal aspirate	19 (22.1)	26 (28.9)	0.302
Bacteremia/blood stream infection	17 (19.8)	15 (16.7)	0.594
Any antibiotics	42 (48.8)	36 (40.0)	0.238
Prophylactic antibiotics	32 (37.2)	50 (55.6)	0.015
Listed for LT	14 (16.3)	28 (31.1)	0.021

Data are presented as median (IQR) or n (% of available data). ∗Missing data on hepatic encephalopathy (HE) grade (according to West Haven criteria) during the first 7 days of study admission: TFS group, n = 2; non-TFS group, n = 1. Mann-Whitney *U* test for continuous variables, Chi-square test for categorical variables. ALF, acute liver failure; APAP, acetaminophen, DILI, drug-induced liver injury; HE, hepatic encephalopathy; ICP, intracranial pressure; KCC, King’s College Criteria; LT, liver transplant; TFS, transplant-free survival.

### Clinical parameters measured on day 1

Comparison of clinical and biochemical parameters at study admission (Day 1) is presented in Table 2, with normalized levels (xULN) of RIPK3, MLKL, GSDMD, and GSDME shown in Table S1. Patients with TFS status were less likely to be on organ support or to achieve grade 3 or 4 HE, and they had lower admission MELD scores than patients with non-TFS status. Serum INR and levels of bilirubin, creatinine, and lactate were lower in patients with TFS than in non-TFS status.

**Table 2:** Clinical and biochemical parameters measured on Day 1.

	Spontaneous alive at Day 21 (TFS, n = 86)		Dead/LT at or before Day 21 (non-TFS, n = 90)		*p* values
	N	n (%)/median (IQR)	N	n (%)/median (IQR)	
Organ support					
Mechanical ventilation	86	36 (41.9)	90	65 (72.2)	<0.001
Vasopressors use	86	16 (18.6)	90	38 (42.2)	<0.001
Renal replacement therapy	86	17 (19.8)	90	32 (35.6)	0.020
MELD	84	29 (23–32.5)	87	36 (29–42)	<0.001
HE coma grade 3 or 4∗	83	36 (43.4)	88	57 (64.8)	0.005
Biochemistry					
Hemoglobin (g/dl)	86	10.8 (9.2–12.7)	90	10.6 (29.3–12.3)	0.885
White blood count (10^9^/L)	85	8.8 (6.3–12.0)	90	11.9 (9.0–16.7)	<0.001
Neutrophils (10^9^/L)	54	83 (73–90)	63	88.5 (81–94)	0.021
Lymphocytes (10^9^/L)	59	9.0 (5.4–14.0)	69	7.0 (4.0–13.0)	0.206
Monocytes (10^9^/L)	60	3.6 (1.0–6.9)	68	2.0 (1.0–5.5)	0.064
Platelet count (10^9^/L)	85	132 (92–187)	90	119 (71–165)	0.176
INR	85	2.7 (1.9–3.8)	87	3.5 (2.5–4.9)	<0.001
ALT (IU/L)	86	3,666.5 (1,690–5,637)	90	2,744.5 (1,292–5,223)	0.166
Bilirubin (mg/dl)	85	4.4 (2.5–6.6)	90	5.6 (3.7–9.6)	<0.001
Ammonia (venous) (μmol/L)	45	89 (57–110)	31	122 (83–293)	0.005
Creatinine (mg/dl)	86	1.5 (0.82–2.9)	90	2.3 (1.2–3.8)	0.006
Lactate (mmol/L)	58	2.9 (1.9–4.7)	68	7.2 (4.0–11.1)	<0.001
Phosphate (mg/dl)	77	2.4 (1.7–3.3)	79	3.4 (2.3–4.8)	<0.001
RIPK3 (ng/ml)	84	6.79 (4.57–9.46)	89	10.29 (6.36–15.30)	<0.001
MLKL (ng/ml)	86	36.23 (29.80–46.60)	89	45.40 (36.33–51.07)	<0.001
GSDMD (ng/ml)	86	2.46 (1.46–3.04)	89	2.64 (1.75–3.97)	0.074
GSDME (ng/ml)	86	2.02 (0.85–3.84)	89	2.80 (1.15–4.72)	0.135

∗ According to West Haven criteria. Mann-Whitney *U* test for continuous variables, Chi-square test for categorical variables. ALT, alanine aminotransferase; GSDMD, gasdermin D; GSDME, gasdermin E; HE, hepatic encephalopathy; INR, international normalized ratio; LT, liver transplant; MELD, model for end-stage liver disease; MLKL, mixed lineage kinase domain-like protein; RIPK3, receptor-interacting protein kinase 3; TFS, transplant-free survival.

Patients with TFS status had significantly lower Day 1 levels of RIPK3 (6.79 *vs*. 10.29 ng/ml) and MLKL (36.23 *vs*. 45.40 ng/ml, *p* <0.001 for both) than patients with non-TFS status (Table 2 and Fig. S2). Levels of RIPK3 and MLKL were also significantly lower in healthy controls than in patients with ALF (Table S2). For Day 1 levels of GSDMD and GSDME, no significant differences were noted between healthy controls and patients with ALF, or between patients with and without TFS status.

### Clinical parameters measured on day 4

Parameters measured on Day 4 (Table 3, Table S1, and Fig. S2) were similar to those observed earlier on Day 1. Patients with TFS status remained less likely to require organ support or to develop grade 3 or 4 HE, with lower MELD scores and reduced serum INR, bilirubin, creatinine, and lactate levels compared with patients with non-TFS status.

**Table 3:** Clinical and biochemical parameters measured on Day 4.

	Spontaneous alive at Day 21 (TFS, n = 86)		Dead/LT at or before Day 21 (non-TFS, n = 90)		*p* value
	N	n (%)/median (IQR)	N	n (%)/median (IQR)	
Organ support					
Mechanical ventilation	86	37 (43.0)	90	82 (91.1)	<0.001
Vasopressors	86	15 (17.4)	90	53 (58.9)	<0.001
Renal replacement therapy	86	22 (25.6)	90	46 (51.1)	<0.001
MELD	78	22 (17–28)	56	32 (25-40)	<0.001
HE coma grade 3 or 4∗	65	33 (50.8)	86	76 (88.4)	<0.001
Biochemistry					
Hemoglobin (g/dl)	82	10.1 (8.8–11.0)	62	9.7 (8.6–10.9)	0.662
White blood count (10^9^/L)	81	S8.6 (6.2–12.8)	62	10.9 (7.0–15.0)	0.107
Neutrophils (10^9^/L)	24	70.6 (60.5–78.8)	17	78 (73–84)	0.078
Lymphocytes (10^9^/L)	26	14.1 (10.0–21.4)	18	8.3 (4.0–16.0)	0.012
Monocytes (10^9^/L)	26	10.5 (6.3–14.5)	18	7.0 (2.9–13.0)	0.181
Platelet count (10^9^/L)	82	97.5 (59–161)	62	70.5 (44–109)	0.006
INR	82	1.55 (1.30–2.00)	59	2.3 (1.7–3.4)	<0.001
ALT (IU/L)	83	1,207 (718–1,960)	60	775 (362.5–1,730.5)	0.063
Bilirubin (mg/dl)	82	6.1 (2.7–10.1)	60	12.3 (7.8–16.3)	<0.001
Ammonia (venous) (μmol/L)	22	57 (45–75)	15	95 (64–133)	0.013
Creatinine (mg/dl)	83	1.04 (0.6–2.1)	60	1.75 (1.24–3.11)	<0.001
Lactate (mmol/L)	19	1.9 (1.3–2.9)	29	3.2 (2.5–5.0)	<0.001
Phosphate (mg/dl)	54	2.7 (2.3–3.4)	30	3.3 (2.6–4.1)	0.053
RIPK3 (ng/ml)	85	7.28 (4.44–11.33)	53	8.00 (3.83–12.83)	0.947
MLKL (ng/ml)	85	16.13 (13.36–17.78)	53	19.10 (16.92–20.47)	<0.001
GSDMD (ng/ml)	85	2.40 (1.68–4.57)	53	2.17 (1.49–4.39)	0.676
GSDME (ng/ml)	84	0.67 (0.29–1.38)	53	1.70 (0.76–2.47)	<0.001

∗ According to West Haven criteria. Mann-Whitney *U* test for continuous variables, Chi-square test for categorical variables. ALT, alanine aminotransferase; GSDMD, gasdermin D; GSDME, gasdermin E; HE, hepatic encephalopathy; INR, international normalized ratio; LT, liver transplant; MELD, model for end-stage liver disease; MLKL, mixed lineage kinase domain-like protein; RIPK3, receptor-interacting protein kinase 3; TFS, transplant-free survival.

TFS group had lower Day 4 levels of MLKL (16.13 *vs*. 19.10 ng/ml, *p* <0.001), but no significant difference in RIPK3 levels (7.28 *vs*. 8.00 ng/ml, *p* = 0.947) compared with non-TFS group. GSDME levels were significantly lower in TFS than in non-TFS group (0.68 *vs*. 1.70 ng/ml, *p* <0.001), whereas GSDMD levels did not significantly differ.

### RIPK3, MLKL, GSDMD, and GSDME in association with 21-day TFS

The associations of RIPK3, MLKL, GSDMD, and GSDME with 21-day TFS were analyzed separately at two time points: on Day 1 (admission) and Day 4, with results presented in Table 4 and Table S3.

**Table 4:** Day 1 and Day 4 levels of RIPK3, MLKL, GSDMD, and GSDME in association with 21-day TFS.

	Unadjusted			Adjusted∗		
	TFS/N	OR (95% CI)†	*p* values	TFS/N	OR (95% CI)†	*p* values
Day 1						
RIPK3	84/173	1.09 (1.04–1.15)	0.001	82/168	1.10 (1.03–1.17)	0.004
MLKL	86/175	1.05 (1.02–1.08)	0.001	84/170	1.01 (0.98–1.05)	0.509
GSDMD	86/175	1.14 (0.96–1.34)	0.133	84/170	1.19 (0.98–1.44)	0.077
GSDME	86/175	1.08 (0.96–1.20)	0.189	84/170	0.99 (0.86–1.15)	0.895
Day 4						
RIPK3	85/138	1.02 (0.98–1.05)	0.426	77/123	0.99 (0.93–1.06)	0.787
MLKL	85/138	1.42 (1.22–1.64)	<0.001	77/123	1.14 (0.93–1.41)	0.202
GSDMD	85/138	1.00 (0.96–1.04)	0.941	77/123	0.98 (0.92–1.05)	0.633
GSDME	84/137	1.32 (1.09–1.60)	0.005	77/123	1.00 (0.88–1.14)	0.983

‡Univariable and multivariable logistic regression, boot strapping, Wald test). APAP, acetaminophen, DILI, drug-induced liver injury; GSDMD, gasdermin D; GSDME, gasdermin E; MLKL, mixed lineage kinase domain-like protein; OR, odds ratio; RIPK3, receptor-interacting protein kinase 3; TFS, transplant-free survival.

∗ Adjusted for etiology (APAP or DILI), MELD, vasopressors use, need for renal replacement therapy and mechanical ventilation, all recorded on the same day as the biomarker measurement (Day 1 or Day 4); degrees of freedom = 6.

† Per 1 ng/ml decrease.

Lower Day 1 levels of RIPK3 were significantly associated with 21-day TFS (odds ratio [OR] 1.09 per 1 ng/ml decrease, 95% CI 1.04–1.15). This association remained after adjusting for RRT and other clinically significant covariates (OR 1.10 per 1 ng/ml decrease, 95% CI 1.03–1.17) and persisted in patients off RRT with further adjustment for serum creatinine (OR 1.08 per 1 ng/ml decrease, 95% CI 1.01–1.16, *p* = 0.028; n = 123), consistent with an effect beyond impaired renal clearance. Lower Day 1 MLKL levels were associated with 21-day TFS in the unadjusted analysis but were non-significant after covariate adjustment. No significant associations were observed between Day 1 levels of GSDMD or GSDME and 21-day TFS.

In unadjusted analyses, lower Day 4 levels of MLKL and GSDME were associated with 21-day TFS. However, these associations were not observed after adjusting for clinically significant covariates. Day 4 RIPK3 and GSDMD levels showed no significant association with 21-day TFS. Adjusted ORs for all biomarkers on Day 1 and Day 4 were largely unchanged in bootstrap resampling (1,000 iterations; Table S4), confirming the stability of effect estimates.

FAMD applied to admission clinical variables for all ALFSG patients with ALF explained 48.2% of the total variance across five dimensions (Table S5), highlighting substantial patient heterogeneity. K-means clustering based on these dimensions identified two subgroups (Table S6): cluster 1 showed lower use of organ support, lower coma grades, and milder laboratory abnormalities, whereas cluster 2 had higher levels across these variables, reflecting more severe physiological derangement at admission. Cluster-specific analyses showed that Day 1 RIPK3 was significantly associated with 21-day TFS in cluster 2 (adjusted OR 1.13 per 1 ng/ml decrease, 95% CI 1.03–1.27), whereas no significant associations were found for cluster 1 or for other biomarkers (Table S7). GMM clustering using FAMD dimensions yielded consistent results (Tables S8 and S9), with low classification uncertainty; only 4% of patients had a maximum posterior probability below 0.7. Together, these findings suggest that the prognostic relevance of RIPK3 is more pronounced in patients with greater clinical severity.

### Performance of day 1 RIPK3 and MLKL in predicting TFS

Given the significant associations between Day 1 levels of RIPK3 and MLKL and 21-day TFS, we further explored the incremental benefit of adding RIPK3 and MLKL to established clinical models (KCC, MELD, and the ALFSG index) for predicting TFS.

**Fig. 1:**
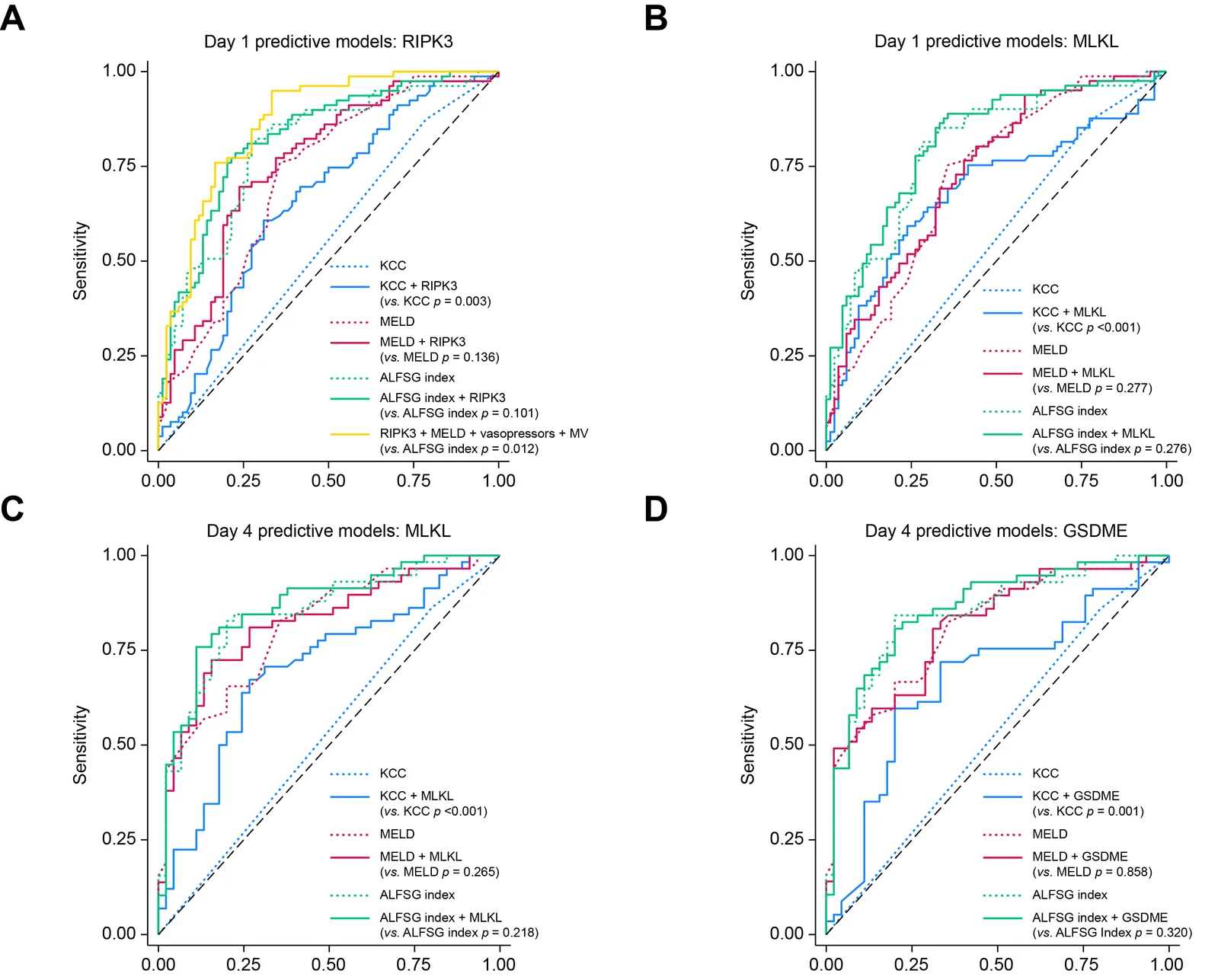
ROC curves for predictive models. ROC curves for predictive models assessed on Day 1 with and without (A) RIPK3 and (B) MLKL, and assessed on Day 4 with and without (C) MLKL and (D) GSDME. The *p* values are derived from pairwise comparison of ROC curves using DeLong tests. AUROC values and 95% CIs are in Table S10. ALFSG, Acute Liver Failure Study Group; AUROC, area under the ROC curve; GSDME, gasdermin E; KCC, King’s College Criteria; MELD, model for end-stage liver disease; MLKL, mixed lineage kinase domain-like protein; RIPK3, receptor-interacting protein kinase 3; ROC, receiver operator curve.

When assessed at admission (Day 1), the AUROC values were 0.57 (95% CI 0.51–0.63) for KCC, 0.72 (95% CI 0.64–0.80) for MELD, and 0.80 (95% CI 0.73–0.87) for the ALFSG index. As shown in Fig. 1 and Table S10, adding RIPK3 and MLKL to KCC improved the AUROC to 0.67 (95% CI 0.59–0.75) and 0.70 (95% CI 0.62–0.78), respectively, significantly outperforming KCC alone (*p* ≤0.003 for both). No significant improvement was observed when RIPK3 or MLKL were added to MELD or ALFSG index.

We conducted a backward selection of covariates listed in Table 2, along with RIPK3 (all measured on Day 1), in association with 21-day TFS. The final model, comprising RIPK3 (OR 1.09 per 1 ng/ml decrease, 95% CI 1.03–1.16), MELD (OR 1.09 per 1 point decrease, 95% CI 1.04–1.15), the use of vasopressors (OR 0.33, 95% CI 0.14–0.77), and MV (OR 0.15, 95% CI 0.05–0.43), achieved an AUROC of 0.87 (95% CI 0.81–0.92), significantly outperforming the ALFSG index (*p* = 0.012; Fig. 1A). In 10-fold cross-validation, the model’s mean AUROC was 0.85 (95% CI 0.77–0.89), indicating good discriminative ability and generalizability (Table S11).

### Performance of day 4 MLKL and GSDME in predicting TFS

When measured on Day 4, the AUROC values were 0.57 (95% CI 0.51–0.63) for KCC, 0.82 (95% CI 0.74–0.89) for MELD, and 0.84 (95% CI 0.77–0.91) for ALFSG index. Day 4 levels of MLKL and GSDME were associated with 21-day TFS (Table 4) and, in our further analysis, led to a significant improvement in AUROC when added to KCC, compared with KCC alone (Fig. 1 and Table S10). However, adding MLKL and GSDME did not result in any significant improvement over MELD or ALFSG index alone.

The performance of all predictive models, assessed on Day 1 or Day 4, remained consistent during cross-validation, with the mean AUROC values for each model presented in Table S11.

### Secondary analysis: temporal changes in RIPK3, MLKL, GSDMD, and GSDME and 21-day TFS

As a secondary mechanistic analysis (Table S12), we examined whether changes in RIPK3, MLKL, GSDMD, and GSDME between Day 1 and Day 4 (Δ = Day 4 – Day 1) were associated with 21-day TFS. In models adjusted for Day 1 levels, greater reductions in MLKL (adjusted OR 1.47 per 1 ng/ml larger decrease, 95% CI 1.23–1.75, *p* <0.001) and GSDME (OR 1.30, 95% CI 1.07–1.59, *p* = 0.010) were associated with higher TFS, whereas changes in RIPK3 and GSDMD were not significant. After further adjustment for clinical covariates, the association remained significant for MLKL (adjusted OR 1.24, 95% CI 1.02–1.52, *p* = 0.035) and borderline for GSDME (OR 1.26, 95% CI 0.99–1.62, *p* = 0.063).

## Discussion

### Key results

This study, as part of the ALFSG, reports a model for predicting 21-day survival without LT using the novel, discovery-phase biomarker RIPK3. Importantly, although the contribution of cell death pathway proteins (necroptosis and pyroptosis) has been extensively studied in pre-clinical models, these are the first data examining these key proteins in patients with ALF. These findings are of great importance in view of the need for accurate prognostic tools to guide use of LT, and the potential to develop novel therapeutic strategies.

The principal findings of this nested case-control study are: (1) an independent association between lower serum RIPK3 levels at the time of admission and 21-day survival in the absence of LT, (2) a novel predictive model of serum RIPK3 that outperforms the ALFSG index, and (3) improved performance of KCC following the addition of RIPK3, MLKL, or GSDME.

### Strengths and limitations

This study represents the first systematic evaluation of inflammatory cell death markers in a large, multicenter, ALF patient cohort. The ALFSG registry represents the largest prospectively collected patient cohort for the study of this rare disease, with detailed and prospectively maintained clinical and laboratory data. As such, this nested case-control design represents a unique opportunity to study novel cell death markers for mechanistic and biomarker purposes. Additionally, samples were collected at two time points, allowing for a dynamic evaluation of biomarkers, along with comparisons to samples from healthy controls.

There are several limitations to this study that warrant consideration. First, as this is a nested case-control study, the event rate of 21-day TFS is different from that published in other cohort series. Because the true baseline risk is not preserved, our analysis can inform association and discrimination (between TFS and non-TFS) but not absolute risk estimates. The case-control design may also have introduced selection bias because the primary outcome of TFS is automatically unbalanced within the clinical profile of the groups. In an attempt to reduce bias, data was collected prospectively, and, within this specific study design, researchers measuring RIPK3, MLKL, GSDMD, and GSDME were blinded to all clinical and outcome data of the patients. Cross-validation assessed internal model performance. FAMD-based clustering demonstrated that the association between admission RIPK3 levels and TFS was preserved and strongest in patients with greater clinical severity; nevertheless, whether this reflects effect modification by severity or a distinct biological phenotype remains unclear and should be interpreted cautiously. Second, although we adjusted for a range of clinically significant covariates, residual confounding could not be excluded. Admission lactate data were unavailable for 50 patients, and further adjustment for lactate in the multivariable analysis yielded a borderline significant OR of 1.09 (95% CI 1.00–1.17) for RIPK3 in association with 21-day TFS, likely because of the reduced sample size of 119. There may also be confounding due to inconsistencies in transplant listing and organ availability across sites; however, our results are unlikely to be affected, as only one patient in this study received LT. Third, KCC was treated as a dichotomized variable. Although this reflects its established use in clinical practice as a decision-making tool, it may reduce the predictive ability and lead to loss of information given its composite nature. The small number of healthy controls further limits the precision and interpretability of biomarker ULN. Finally, the cohort presented largely reflects APAP-related ALF; therefore, we must be cautious in generalizing these findings to non-APAP etiologies of ALF.

### Comparison with literature

Programmed, non-apoptotic cell death pathways are increasingly recognized to play a crucial role in host response to pathogens and sterile inflammation. Biomarkers for several non-apoptotic pathways (*e.g*. necroptosis, pyroptosis, and ferroptosis) have been found to be elevated in critical illness and associated with organ dysfunction. The necroptosis marker RIPK3 is reportedly elevated in critically ill patients with sepsis and associated with higher mortality.^35^ Similarly, the pyroptosis marker GSDMD was elevated in critically ill patients with acute respiratory distress syndrome.^36^ From a mechanistic perspective, necroptosis and pyroptosis are lytic, pro-inflammatory pathways that promote the release of intracellular DAMPs. Pre-clinical data suggest this lytic cell death plays an important role in mounting an immune response against infectious insults, particular intracellular pathogens.^36^ However, these pathways are increasingly thought to contribute to aberrant tissue injury and adverse outcomes in sepsis and chronic inflammatory disease, although causal evidence is limited to pre-clinical models.

In the context of liver disease, non-apoptotic cell death markers have been associated with both acute and chronic liver injury. In ALF, Kondo *et al.*^37^ recently demonstrated an association of serum RIPK3 levels with mortality in a cohort of 71 patients with acute liver injury, including 11 with ALF; no variables were found to significantly predict survival on multivariable analysis, likely because of the small size of the cohort. From a mechanistic perspective the situation is more complex, and there has been controversy surrounding the role of the RIPKs (RIPK1 and RIPK3) and the occurrence of necroptosis in pre-clinical APAP-ALF models.^17^^,^^22^^,^^38^ Hepatocytes do not express RIPK3 at baseline, although immune cells and liver endothelial cells express abundant amounts of RIPK3; it is therefore likely that elevated circulating levels of RIPK3 in ALF reflect systemic inflammatory injury and regulated necrosis rather than hepatocyte-specific necroptosis alone.[39], [40], [41] Further work is required to fully delineate the cellular origin of RIPK3 in acute liver injury.

Phosphorylated MLKL (p-MLKL) is the effector protein of necroptotic cell death, causing membrane pore formation and lytic cell death.^42^ In this study, we measured total MLKL rather than p-MLKL. Nevertheless, MLKL levels were consistently lower in TFS than in non-TFS group, likely a reflection of MLKL being downstream in the necroptosis pathway. Greater decline in MLKL levels between Day 1 and Day 3 was also associated with TFS, possibly reflecting the resolution of necroptosis and recovery. However, we acknowledge that although these associations are intriguing, we cannot make firm mechanistic conclusions regarding the role of necroptosis in the absence of p-MLKL measurement.

The data presented here do not support an association between GSDMD or GSDME levels and outcome in ALF. This suggests the pyroptosis pathway is not a major effector of systemic inflammation in acute liver injury. This is likely a different situation to patients with established cirrhosis and superimposed multiorgan failure (acute-on-chronic liver failure [ACLF]), where chronic injury, immune priming, and the altered hepatic microenvironment may sensitize lytic cell death pathways. Consistent with this, our own data and that of others demonstrate upregulation of the hepatocyte caspase-4/GSDMD pathway in fibrosis and steatohepatitis, thus supporting increased susceptibility to pyroptosis in chronic liver disease. Pre-clinical models further support a causal role for GSDMD in the development of ACLF.^43^^,^^44^ Nevertheless, the situation in ACLF is likely more complex and may depend on etiology and precipitating event. Recently, Verma *et al.*^45^ presented a biomarker study exploring RIPK3 and GSDMD in patients with decompensated cirrhosis or ACLF, predominantly with alcohol-related liver disease. This group found elevations of RIPK3 and GSDMD, although RIPK3 levels provided the strongest prognostic discrimination. Conversely, Zhao *et al.*^46^ recently provided mechanistic evidence of GSDMD-mediated pyroptosis in hepatitis B-related ACLF. This group used unbiased multi-omic methods to demonstrate that pyroptosis is the dominant liver cell death pathway, and *ex vivo* liver function could be improved with antipyroptosis therapy.

From a translational perspective, there is currently no Good Laboratory Practice-validated assay for RIPK3, and therefore integration into transplantation decision-making pathways is not yet feasible. Therefore, RIPK3 remains a discovery-phase biomarker, although the results presented here support the development of an assay suitable for regulatory approval, and prospective studies of RIPK3 as a clinical ALF biomarker.

## Conclusions

Serum levels of the necroptosis mediator RIPK3 are associated with TFS in ALF. GSDMD and GSDME levels were not associated with outcome in ALF. These data support validation studies of RIPK3 as a mechanistic biomarker in ALF, and further translational work of RIPK3 inhibition as a potential treatment modality for ALF.

## Abbreviations

ACLF, acute-on-chronic liver failure; ALF, acute liver failure; ALFSG, Acute Liver Failure Study Group; ALT, alanine aminotransferase; APAP, acetaminophen; DAMPs, damage-associated molecular patterns; DILI, drug-induced liver injury; FAMD, factor analysis of mixed data; GMM, Gaussian mixture model; GSDMD, gasdermin D; GSDME, gasdermin E; HE, hepatic encephalopathy; ICP, intracranial pressure; INR, international normalized ratio; KCC, King’s College Criteria; LT, liver transplantation; MELD, model for end-stage liver disease; MLKL, mixed lineage kinase domain-like; MV, mechanical ventilation; OR, odds ratio; p-MLKL, phosphorylated MLKL; RIPK3, receptor-interacting protein kinase 3; ROC, receiver operator curve; RRT, renal replacement therapy; TFS, transplant-free survival; TNF, tumor necrosis factor; ULN, upper limit of normal; VIF, variance inflation factor.

## Authors’ contributions

Study conception and design: GM, CJK. Laboratory analysis: US. Data analysis: GM, CJK. Data interpretation: GM, CD, JLS, CJK. Statistical analysis: CD, JLS, VD. Dataset preparation: SB, VD. Drafted the final manuscript: GM, CJK. Critical revision of the final manuscript: CD, JLS, SB, LD, VD, WML. Supervision of entire US Acute Liver Failure Study Group (U-01 Grant): WML.

## Data availability

Data presented in this study are available on application to the ALFSG.

## Financial support

The study was sponsored by 10.13039/100000002National Institutes of Health grant U-01 58369 from the National Institute of Diabetes, Digestive and Kidney Diseases.

## Conflicts of interest

GM has shares in Hepyx Ltd and Yaqrit Ltd. CJK reports receiving consulting fees from Genfit, Vantive, Mallinckrodt Pharmaceuticals, Morphocell Inc., and DSMB committee service for Miromatrix. All other authors have no personal or funding conflicts of interest. Please refer to the accompanying ICMJE disclosure forms for further details.
