# Supplementary material for: Serum receptor-interacting protein kinase 3 (RIPK3) is associated with transplant-free survival in acute liver failure
Source: JHEP Rep. 2026 Aug 4;8(10):101962. doi: 10.1016/j.jhepr.2026.101962 (PMC13571404; doi:10.1016/j.jhepr.2026.101962)
Supplement: Supplementary file 1 [file mmc1.pdf]

Serum receptor-interacting protein kinase 3 (RIPK3) is associated with transplant-free survival in acute liver failure

Gautam Mehta, Chengyi Ding, Ugo Soffientini, Jaime L. Speiser, Sarah Bahnassi, Lily Dara, Valerie Durkalski, William M. Lee, Constantine J. Karvellas, for the US Acute Liver Failure Study Group

Table of contents

Supplementary Figures ……………………………………………………………………3

Supplementary Tables……………………………………………………………..……………5

Fig. S1: Patient selection for the nested case-control analysis

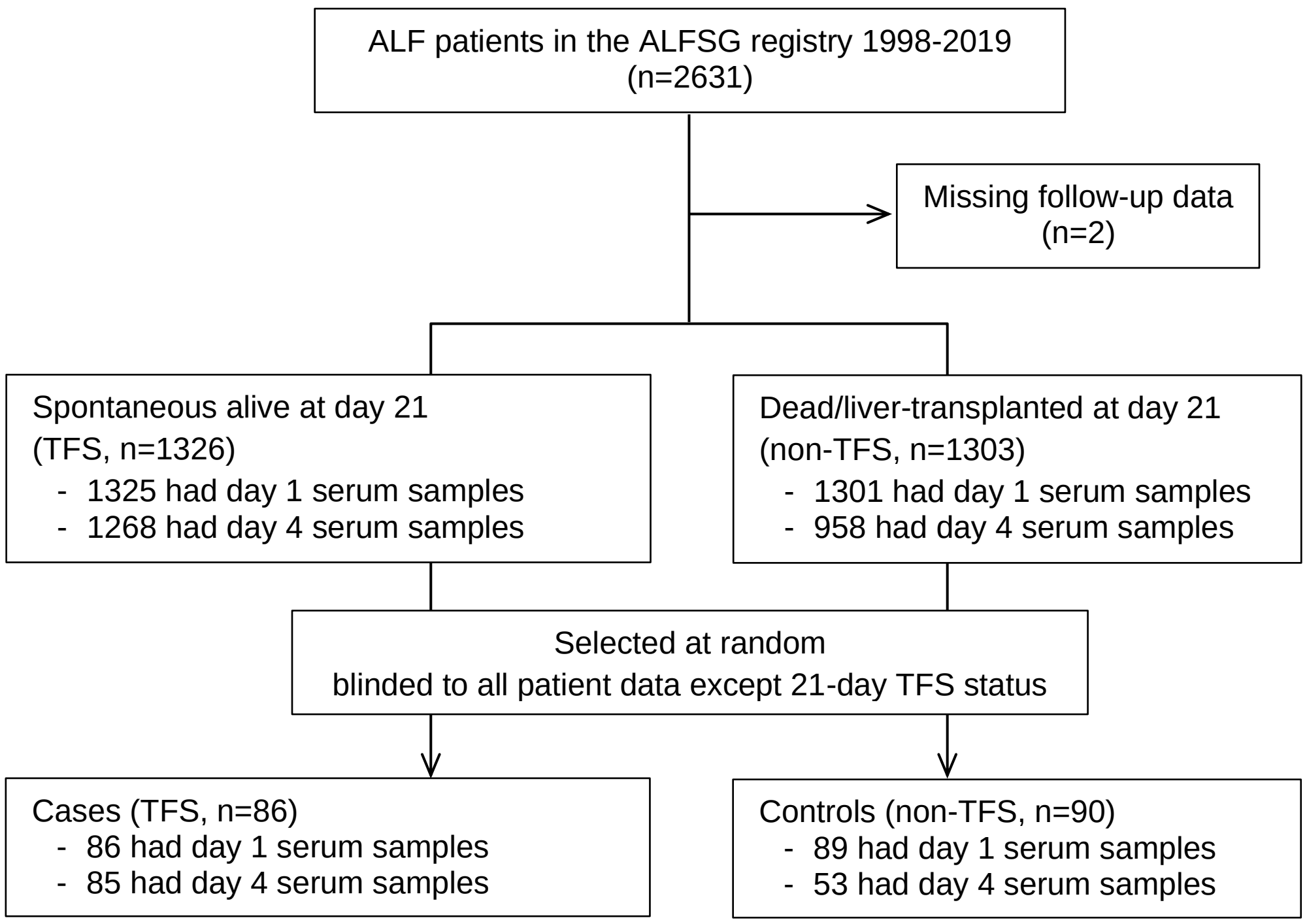

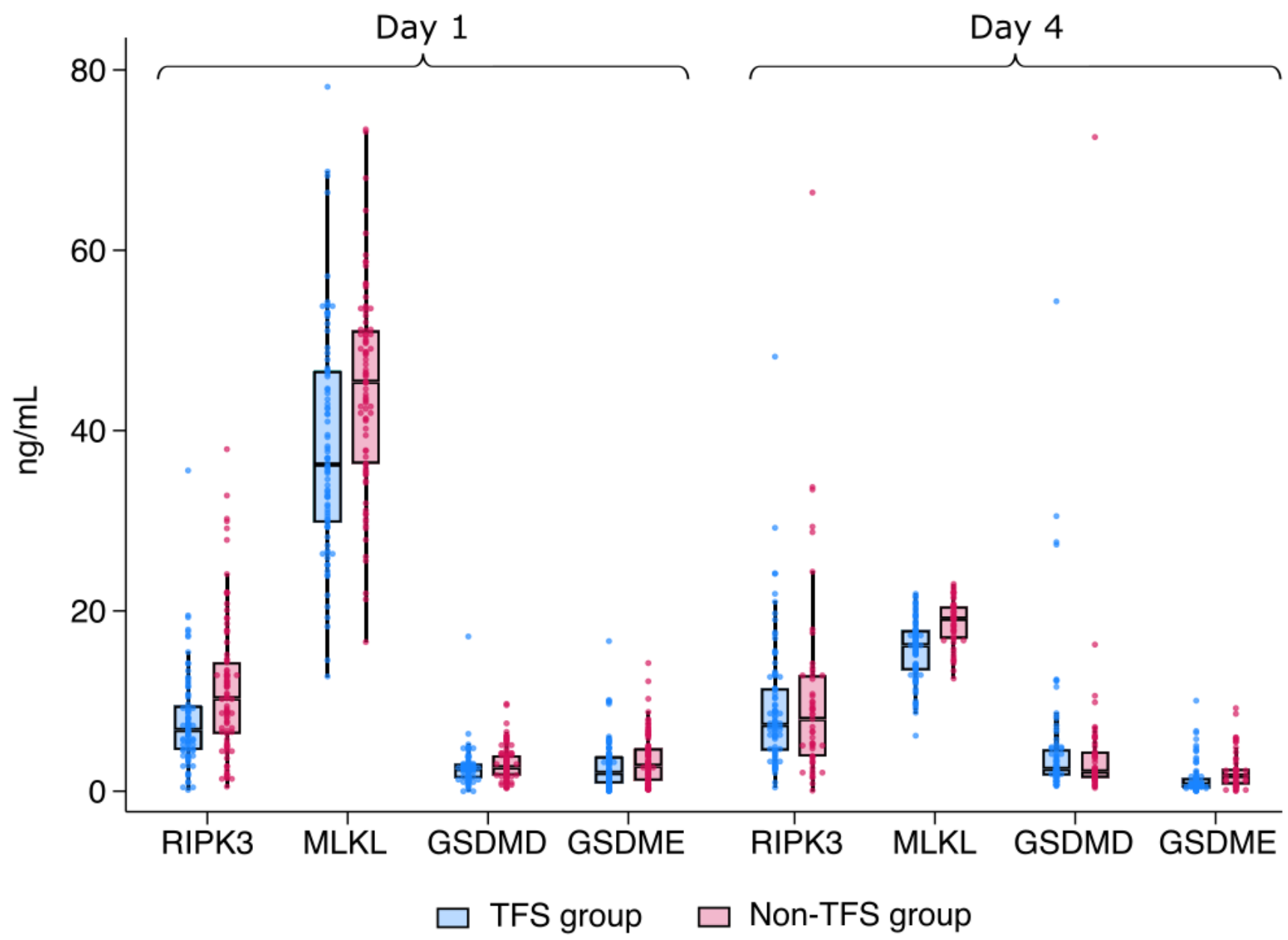

Fig. S2: Day 1 and day 4 levels of RIPK3, MLKL, GSDMD and GSDME in 176 ALF patients

Table S1: RIPK3, MLKL, GSDMD, and GSDME levels (xULN) by 21-day TFS status

	Spontaneous alive at day 21 (TFS, n=86)		Dead/LT at or before day 21 (non-TFS, n=90)		p value
	N	n (%)/median (IQR)	N	n (%)/median (IQR)	
**Day 1**					
RIPK3 xULN	84	2.46 (1.66, 3.43)	89	3.73 (2.31, 5.18)	<0.001
MLKL xULN	86	10.14 (8.34, 13.04)	89	12.71 (10.17, 14.29)	<0.001
GSDMD xULN	86	1.01 (0.60, 1.24)	89	1.08 (0.72, 1.62)	0.074
GSDME xULN	86	0.18 (0.08, 0.34)	89	0.25 (0.10, 0.42)	0.135
**Day 4**					
RIPK3 xULN	85	2.64 (1.61, 4.11)	53	2.90 (1.39, 4.66)	0.947
MLKL xULN	85	4.51 (3.74, 4.97)	53	5.35 (4.73, 5.73)	<0.001
GSDMD xULN	85	0.98 (0.69, 1.87)	53	0.89 (0.61, 1.79)	0.676
GSDME xULN	84	0.06 (0.03, 0.12)	53	0.15 (0.07, 0.22)	<0.001

Normalised as multiples of the ULN (upper limit of normal), based on the maximum value observed in healthy controls (Mann-u-Whitney).

Table S2: Demographics and serum levels of RIPK3, MLKL, GSDMD, and GSDME in healthy controls

	Healthy controls (n=8)
Age (years)	44.5 (44-52)
Male	3 (37.5)
White/Caucasian	8 (100)
RIPK3 (ng/mL)	2.01 (1.57-2.18)
MLKL (ng/mL)	2.86 (2.33-3.26)
GSDMD (ng/mL)	1.65 (1.38-1.87)
GSDME (ng/mL)	2.33 (0.13-6.12)

Data are median (interquartile range) or n (%).

Table S3: RIPK3, MLKL, GSDMD, and GSDME levels (xULN) in association with 21-day TFS

	TFS	N	Adjusted OR (95%CI) *	p value
**Day 1**				
RIPK3 xULN	82	168	1.30 (1.09-1.54)	0.004
MLKL xULN	84	170	1.04 (0.92-1.19)	0.509
GSDMD xULN	84	170	1.52 (0.96-2.43)	0.077
GSDME xULN	84	170	0.89 (0.17-4.67)	0.895
**Day 4**				
RIPK3 xULN	77	123	0.98 (0.81-1.17)	0.787
MLKL xULN	77	123	1.61 (0.77-3.37)	0.202
GSDMD xULN	77	123	0.96 (0.82-1.13)	0.633
GSDME xULN	77	123	1.02 (0.24-4.30)	0.983

* Adjusted for aetiology (APAP or DILI), MELD, vasopressors use, need for renal replacement therapy and mechanical ventilation, all recorded on the same day as the biomarker measurement (day 1 or day 4); per 1x ULN (upper limit of normal) decrease. (Univariate logistic regression)

Table S4: Association of RIPK3, MLKL, GSDMD, and GSDME levels with 21-day TFS, estimated using 1,000 bootstrap iterations

	Adjusted OR (95%CI) *	p value
**Day 1**		
RIPK3	1.10 (1.02-1.19)	0.018
MLKL	1.01 (0.97-1.06)	0.570
GSDMD	1.19 (0.96-1.47)	0.110
GSDME	0.99 (0.85-1.15)	0.899
**Day 4**		
RIPK3	0.99 (0.91-1.08)	0.827
MLKL	1.14 (0.91-1.44)	0.258
GSDMD	0.98 (0.87-1.12)	0.808
GSDME	1.00 (0.78-1.28)	0.991

* Adjusted for aetiology (APAP or DILI), MELD, vasopressors use, need for renal replacement therapy and mechanical ventilation, all recorded on the same day as the biomarker measurement (day 1 or day 4); per 1 ng/mL decrease. (logistic regression)

Table S5: Contributions of admission clinical variables to FAMD dimensions

Variable	Dim 1 (%)	Dim 2 (%)	Dim 3 (%)	Dim 4 (%)	Dim 5 (%)
Age (years)	0.05	1.48	4.47	1.42	0.14
Hemoglobin (g/dL)	6.67	15.81	5.85	7.79	2.96
White Blood count (10^9/L)	1.20	1.34	10.33	0.05	23.88
Lymphocytes (10^9/L)	3.32	4.13	0.33	0.91	5.19
Platelet count (10^9/L)	2.69	1.44	1.39	4.34	22.95
ALT (IU/L)	2.24	20.94	5.19	1.22	0.70
AST (IU/L)	5.89	16.32	2.05	3.60	0.02
Bilirubin (mg/dL)	3.03	5.05	15.38	0.37	0.21
Creatinine (mg/dL)	8.35	0.01	5.51	4.58	4.69
Phosphate (mg/dL)	5.57	0.02	21.94	4.65	0.29
PH	5.82	1.58	5.79	7.44	1.87
Hematocrit	6.23	16.89	6.03	7.00	1.90
INR	0.51	7.10	0.18	1.62	2.33
HE coma grade	9.20	1.11	0.05	25.98	0.03
Male	0.08	1.17	7.52	0.35	27.29
Mechanical ventilation	12.42	0.71	0.27	22.17	0.01
Vasopressors use	12.86	0.06	0.98	3.60	0.03
Race	0.33	2.71	1.46	1.68	3.38
Aetiology (APAP/DILI)	1.46	1.54	4.53	0.16	0.22
Renal replacement therapy	12.06	0.59	0.75	1.08	1.93

Table S6: Admission characteristics of FAMD-based k-means clusters in ALFSG ALF patients

	Cluster 1 (n=1599)	Cluster 2 (n=1020)	p value
Age (years)	41 (30-53)	41 (30-53)	0.595
HE coma grade	2 (1-3)	4 (3-4)	<0.001
Biochemistry			
Hemoglobin (g/dL)	11.6 (10.2-13.2)	9.8 (8.7-11.3)	<0.001
White Blood count (10^9/L)	9.5 (6.8-13.7)	11.4 (7.2-17.1)	<0.001
Lymphocytes (10^9/L)	10.8 (5.9-18.0)	7.0 (4.0-11.0)	<0.001
Monocytes (10^9/L)	6.0 (2.5-10.0)	3.0 (1.0-6.0)	<0.001
Platelet count (10^9/L)	144 (96-203)	102 (64-153)	<0.001
INR	2.7 (2.0-4.0)	2.8 (2.0-4.4)	0.016
ALT (IU/L)	1602 (500.5-3786.5)	2640.5 (947-5045)	<0.001
AST (IU/L)	1147.5 (390-3283)	2752 (721.5-7164.5)	<0.001
Bilirubin (mg/dL)	8.9 (3.9-22.1)	5.5 (3.4-10.5)	<0.001
Ammonia (venous) (μmol/L)	88 (57-133)	115 (73-178)	<0.001
Creatinine (mg/dL)	1.1 (0.7-2.0)	2.7 (1.7-4.2)	<0.001
Lactate (mmol/L)	2.9 (1.9-5.0)	5.8 (3.3-10.5)	0.461
Phosphate (mg/dL)	2.7 (1.8-3.7)	3.7 (2.6-5.6)	<0.001
Aetiology			
APAP	1378 (86.2)	961 (94.2)	<0.001
DILI	221 (13.8)	59 (5.8)	
Male	520 (32.5)	305 (29.9)	0.173
Race			
White/Caucasian	1190 (74.4)	782 (76.7)	0.037
African American	254 (15.9)	132 (12.9)	
Other	155 (9.7)	106 (10.4)	
Organ support			
Mechanical ventilation	358 (22.4)	870 (85.3)	<0.001
Vasopressors use	43 (2.7)	509 (49.9)	<0.001
Renal replacement therapy	53 (3.3)	545 (53.4)	<0.001

Data are median (interquartile range) or n (% of available data). (Mann-U-Whitney for continuous variables, chi-squared for categorical)

Table S7: K-means cluster-specific associations of RIPK3, MLKL, GSDMD, and GSDME levels on day 1 with 21-day TFS

	Cluster 1 (TFS=54/n=86)		Cluster 2 (TFS=32/n=90)	
	Adjusted OR (95%CI) *	p value	Adjusted OR (95%CI) *	p value
**Per 1 ng/mL decrease**				
RIPK3	1.04 (0.96-1.12)	0.322	1.13 (1.03-1.27)	0.017
MLKL	1.02 (0.97-1.08)	0.404	1.01 (0.96-1.06)	0.805
GSDMD	1.12 (0.89-1.41)	0.314	1.38 (0.99-2.10)	0.088
GSDME	0.89 (0.72-1.06)	0.220	1.15 (0.91-1.50)	0.253
**Per 1x ULN decrease**				
RIPK3 xULN	1.11 (0.90-1.37)	0.322	1.40 (1.09-1.92)	0.017
MLKL xULN	1.08 (0.90-1.30)	0.404	1.02 (0.86-1.22)	0.805
GSDMD xULN	1.31 (0.75-2.33)	0.314	2.21 (0.97-6.14)	0.088
GSDME xULN	0.26 (0.02-1.95)	0.220	5.07 (0.36-100.62)	0.253

* Adjusted for aetiology (APAP or DILI), MELD, vasopressors use, need for renal replacement therapy and mechanical ventilation, all recorded on day 1.

**Univariable and multivariable logistic regression, Wald test.

Table S8: Admission characteristics of FAMD-based GMM clusters in ALFSG ALF patients

	Cluster 1 (n=1488)	Cluster 2 (n=1131)	p value
Age (years)	42 (30-55)	39 (29-51)	<0.001
HE coma grade	2 (1-2)	4 (3-4)	<0.001
Biochemistry			
Hemoglobin (g/dL)	11.5 (9.9-13.1)	10.2 (9.0-11.8)	<0.001
White Blood count (10^9/L)	9.8 (6.9-14.3)	10.7 (6.8-16.0)	0.011
Lymphocytes (10^9/L)	10.0 (5.0-17.0)	7.9 (4.8-14.0)	0.001
Monocytes (10^9/L)	5.5 (2.0-10.0)	4.0 (1.0-7.1)	<0.001
Platelet count (10^9/L)	134 (89-193)	115 (71-175)	<0.001
INR	2.70 (2.00-3.88)	2.80 (2.00-4.47)	0.001
ALT (IU/L)	1,705 (479.8-4,175.2)	2,310.5 (834-4,443)	0.201
AST (IU/L)	1,406.0 (411.0-4,326.0)	2,106 (582.0-5,718.0)	<0.001
Bilirubin (mg/dL)	8.0 (3.6-21.8)	6.3 (3.6-13.2)	<0.001
Ammonia (venous) (µmol/L)	83 (56-127)	116 (77-177)	<0.001
Creatinine (mg/dL)	1.3 (0.8-2.6)	2.1 (1.1-3.5)	<0.001
Lactate (mmol/L)	3.0 (1.9-5.6)	5.0 (3.0-9.6)	0.218
Phosphate (mg/dL)	3.0 (2.1-4.3)	3.1 (2.1-4.7)	0.010
Aetiology			
APAP	1,278 (85.9)	1,061 (93.8)	<0.001
DILI	210 (14.1)	70 (6.2)	<0.001
Male	488 (32.8)	337 (29.8)	0.111
Race			
White/Caucasian	1,126 (75.7)	846 (74.8)	0.022
African American	229 (15.4)	157 (13.9)	<0.001
Other	133 (8.9)	128 (11.3)	<0.001
Organ support			
Mechanical ventilation	127 (8.5)	1,101 (97.3)	<0.001
Vasopressors use	65 (4.4)	487 (43.1)	<0.001
Renal replacement therapy	116 (7.8)	482 (42.6)	<0.001

Data are median (interquartile range) or n (% of available data).
(Mann-U-Whitney for continuous variables, chi-squared for categorical)

Table S9: GMM cluster-specific associations of RIPK3, MLKL, GSDMD, and GSDME levels on day 1 with 21-day TFS

	Cluster 1 (TFS=54/n=83)		Cluster 2 (TFS=32/n=93)	
	Adjusted OR (95%CI) *	p value	Adjusted OR (95%CI) *	p value
**Per 1 ng/mL decrease**				
RIPK3	1.03 (0.95-1.11)	0.500	1.14 (1.04-1.27)	0.014
MLKL	1.01 (0.96-1.06)	0.739	1.02 (0.97-1.08)	0.488
GSDMD	1.09 (0.85-1.37)	0.448	1.53 (1.05-2.53)	0.054
GSDME	0.92 (0.75-1.10)	0.393	1.08 (0.87-1.40)	0.501
**Per 1x ULN decrease**				
RIPK3 xULN	1.08 (0.86-1.34)	0.500	1.42 (1.10-1.95)	0.014
MLKL xULN	1.03 (0.86-1.23)	0.739	1.07 (0.89-1.30)	0.488
GSDMD xULN	1.23 (0.67-2.17)	0.448	2.83 (1.14-9.68)	0.054
GSDME xULN	0.39 (0.04-2.92)	0.393	2.49 (0.19-44.93)	0.501

* Adjusted for aetiology (APAP or DILI), MELD, vasopressors use, need for renal replacement therapy and mechanical ventilation, all recorded on day 1.

**Univariable and multivariable logistic regression, Wald test.

Table S10: Performance of models in predicting 21-day TFS

	TFS	N	AUROC (95%CI)
**Day 1**			
KCC	86	176	0.57 (0.51, 0.63)
KCC+RIPK3	84	173	0.67 (0.59, 0.75)
KCC+MLKL	86	175	0.70 (0.62, 0.78)
MELD	84	171	0.72 (0.64, 0.80)
MELD+RIPK3	82	168	0.75 (0.68, 0.83)
MELD+MLKL	84	170	0.73 (0.65, 0.80)
ALFSG Index	81	166	0.80 (0.73, 0.87)
ALFSG Index+RIPK3	79	163	0.83 (0.76, 0.89)
ALFSG Index+MLKL	81	165	0.82 (0.75, 0.88)
RIPK3+MELD+Vasopressors use+MV	82	168	0.87 (0.81, 0.92)
**Day 4**			
KCC	86	176	0.57 (0.51, 0.63)
KCC+MLKL	85	138	0.77 (0.69, 0.85)
KCC+GSDME	84	137	0.70 (0.61, 0.79)
MELD	78	134	0.82 (0.74, 0.89)
MELD+MLKL	77	123	0.83 (0.76, 0.91)
MELD+GSDME	76	122	0.81 (0.74, 0.89)
ALFSG Index	60	116	0.84 (0.77, 0.91)
ALFSG Index+MLKL	59	105	0.86 (0.79, 0.93)
ALFSG Index+GSDME	58	104	0.85 (0.78, 0.93)

ALFSG= Acute Liver Failure Study Group, AUROC=area under the ROC curve, KCC= King's College Criteria, MELD= Model for End-stage Liver Disease, MV= mechanical ventilation, TFS=transplant-free survival.

Table S11: Cross-validated predictive performance of models

	mean AUROC (95%CI)	Paired t-test p value		
		vs. KCC	vs. MELD	vs. ALFSG Index
**Day 1**				
KCC	0.57 (0.35, 0.53)	NA		
KCC+RIPK3	0.67 (0.56, 0.73)	<0.001		
KCC+MLKL	0.71 (0.57, 0.75)	0.021		
MELD	0.73 (0.62, 0.78)		NA	
MELD+RIPK3	0.75 (0.65, 0.81)		0.624	
MELD+MLKL	0.74 (0.61, 0.77)		0.884	
ALFSG Index	0.81 (0.70, 0.85)			NA
ALFSG Index+RIPK3	0.81 (0.71, 0.86)			0.959
ALFSG Index+MLKL	0.80 (0.74, 0.88)			0.820
RIPK3+MELD+Vasopressors use+MV	0.85 (0.77, 0.89)			<0.001
**Day 4**				
KCC	0.57 (0.35, 0.53)	NA		
KCC+MLKL	0.76 (0.66, 0.83)	<0.001		
KCC+GSDME	0.70 (0.58, 0.77)	0.008		
MELD	0.83 (0.71, 0.87)		NA	
MELD+MLKL	0.83 (0.72, 0.89)		0.879	
MELD+GSDME	0.81 (0.73, 0.89)		0.516	
ALFSG Index	0.81 (0.73, 0.89)			NA
ALFSG Index+MLKL	0.87 (0.75, 0.92)			0.221
ALFSG Index+GSDME	0.85 (0.78, 0.93)			0.426

ALFSG= Acute Liver Failure Study Group, AUROC=area under the ROC curve, KCC= King's College Criteria, MELD= Model for End-stage Liver Disease, MV= mechanical ventilation, TFS=transplant-free survival.

Table S12: Temporal changes in RIPK3, MLKL, GSDMD, and GSDME in association with 21-day TFS

	TFS	N	OR (95%CI) *	p value
**Adjusted for day 1 level**				
ΔRIPK3 (Δ=day 4-day 1, ng/mL)	83	135	0.99 (0.95, 1.04)	0.725
ΔMLKL	85	137	1.47 (1.23, 1.75)	<0.001
ΔGSDMD	85	137	1.00 (0.96, 1.04)	0.828
ΔGSDME	84	136	1.30 (1.07, 1.59)	0.010
**Adjusted for day 1 level + clinical covariates** **				
ΔRIPK3	81	131	0.99 (0.94, 1.04)	0.559
ΔMLKL	83	133	1.24 (1.02, 1.52)	0.035
ΔGSDMD	83	133	0.99 (0.94, 1.05)	0.859
ΔGSDME	82	132	1.26 (0.99, 1.62)	0.063

* Per 1 ng/mL larger decrease.

** Etiology (APAP or DILI), MELD, vasopressors use, need for renal replacement therapy and mechanical ventilation, all recorded on day 1.

***Logistic regression, Wald test.
