# Supplementary material for: Serum receptor-interacting protein kinase 3 (RIPK3) is associated with transplant-free survival in acute liver failure
Source: JHEP Rep. 2026 Aug 4;8(10):101962. doi: 10.1016/j.jhepr.2026.101962 (PMC13571404; doi:10.1016/j.jhepr.2026.101962)
Supplement: Supplementary file 3 [file mmc4.pdf]

JHEP|Reports

Serum receptor-interacting protein kinase 3 (RIPK3) is associated with transplant-free survival in acute liver failure

Authors

Gautam Mehta, Chengyi Ding, Ugo Soffientini, …, Valerie Durkalski, William M. Lee, Constantine J. Karvellas

Correspondence

dean.karvellas@ualberta.ca (C.J. Karvellas).

Graphical abstract

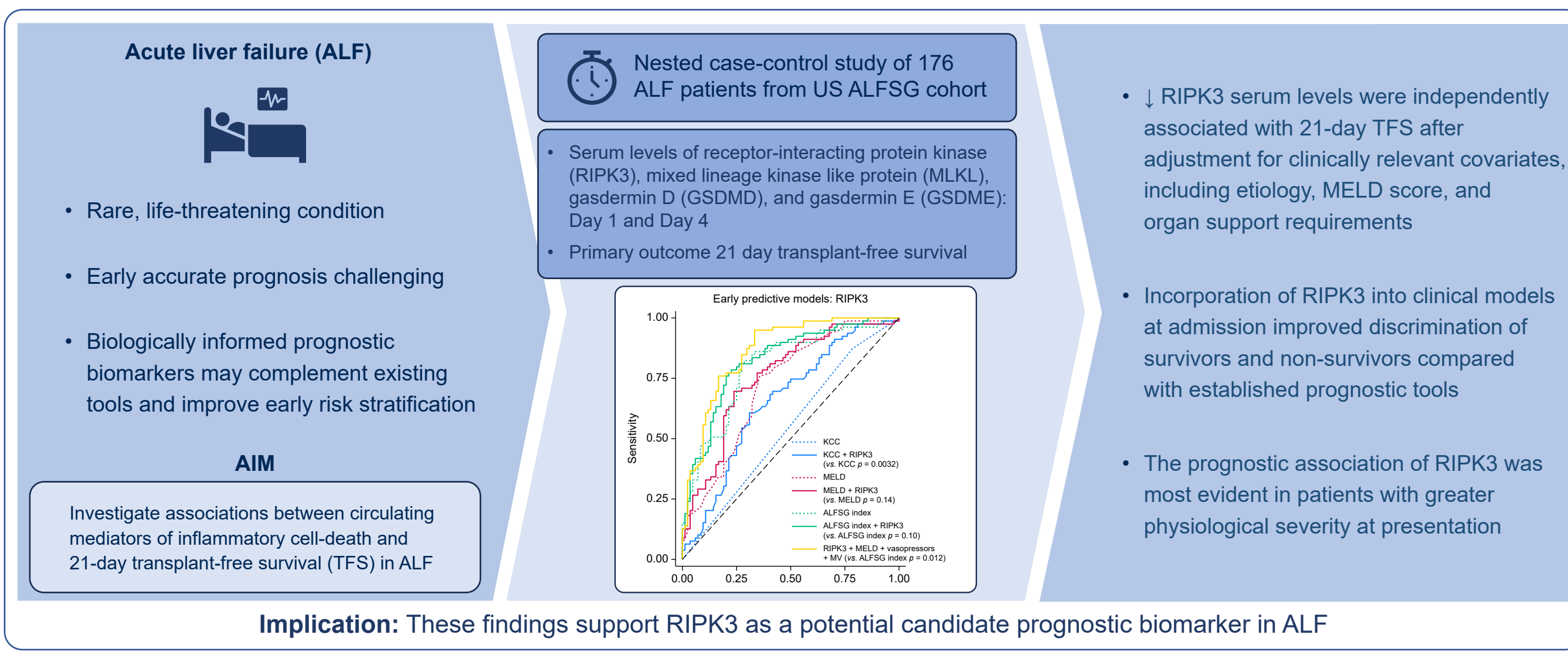

Highlights:

- Prognostication in ALF remains challenging, with commonly used clinical scores demonstrating limited sensitivity.
- Lower serum levels of RIPK3 were independently associated with 21-day transplant-free survival in ALF.
- Incorporating RIPK3 into clinical models at admission improved discrimination of survivors and non-survivors.
- The prognostic association of RIPK3 was most evident in patients with greater physiological severity at presentation.

Impact and implications:

In this study of 176 patients with ALF, lower serum receptor-interacting protein kinase 3 (RIPK3) levels were independently associated with 21-day transplant-free survival. The incorporation of RIPK3 into established prognostic tools improved discrimination between survivors and non-survivors. This study has particular clinical relevance to patients with ALF who had a higher burden of multiorgan failure, in whom the prognostic association of RIPK3 was more evident. Incorporating RIPK3 in current prognostic models may assist clinical decisions regarding which patients with ALF require transplantation *vs*. those who may recover with medical therapy; however, future prospective studies are needed to validate these findings.

https://doi.org/10.1016/j.jhepr.2026.101962

© 2026 The Author(s). Published by Elsevier B.V. on behalf of European Association for the Study of the Liver (EASL). This is an open access article under the CC BY license (http://creativecommons.org/licenses/by/4.0/).

Serum receptor-interacting protein kinase 3 (RIPK3) is associated with transplant-free survival in acute liver failure

Gautam Mehta[1,2,†], **Chengyi Ding**[1,†], Ugo Soffientini[1,2], Jaime L. Speiser[3], Sarah Bahnassi[1], Lily Dara[4], Valerie Durkalski[5], William M. Lee[6], Constantine J. Karvellas[7,*], US Acute Liver Failure Study Group

JHEP Reports **2026**. vol. 8 | 1–10

Check for updates

Background & Aims: Prognostication in acute liver failure (ALF) due to acetaminophen or drug-induced liver injury remains challenging. Biologically informed prognostic biomarkers may complement existing tools and improve early risk stratification. We aimed at investigating the associations between circulating mediators of inflammatory cell death and 21-day transplant-free survival (TFS) in ALF.

Methods: We conducted a nested case-control study of 176 adults with ALF due to acetaminophen or drug-induced liver injury; they were selected from the Acute Liver Failure Study Group registry. Serum levels of receptor-interacting protein kinase 3 (RIPK3), mixed lineage kinase domain-like protein (MLKL), gasdermin D (GSDMD), and gasdermin E (GSDME) were measured at admission (Day 1) and Day 4. Associations with 21-day TFS were evaluated using multivariable logistic regression and receiver operating characteristic analyses. Machine learning methods were used to characterize patient heterogeneity and assess the robustness of biomarker-outcome associations.

Results: At Day 21, 86 patients with ALF were alive without a liver transplant, whereas 90 died or received a liver transplant. Lower admission RIPK3 levels were independently associated with 21-day TFS after adjustment for clinically relevant covariates, including etiology, MELD score, and organ support requirements (adjusted odds ratio 1.10 per 1 ng/ml decrease; 95% CI 1.03–1.17). Incorporation of RIPK3 into clinical models at admission improved discrimination compared with established prognostic tools (AUROC 0.87; 95% CI, 0.81–0.92). The prognostic association of RIPK3 was most evident in patients with greater physiological severity at presentation.

Conclusions: Serum RIPK3 is independently associated with TFS in ALF and improves the discriminative performance of clinical prognostic models. These findings support RIPK3 as a candidate prognostic biomarker in ALF.

© 2026 The Author(s). Published by Elsevier B.V. on behalf of European Association for the Study of the Liver (EASL). This is an open access article under the CC BY license (http://creativecommons.org/licenses/by/4.0/).

Introduction

Acute liver failure (ALF) is defined by the occurrence of hepatic encephalopathy (HE) and synthetic dysfunction within 26 weeks of the first symptoms of liver disease.[1] Currently, the most common cause of ALF in North America is acetaminophen (APAP).[2,3] The most widely used prognostic model in patients with APAP-ALF is the King's College Criteria (KCC).[4,5] Although initially published in 1989, subsequent studies using KCC have shown relatively poor sensitivity of the APAP criteria, ranging 25–76%.[3,6–9] More recently, the Acute Liver Failure Study Group (ALFSG) prognostic index also demonstrated limited sensitivity (41%) in APAP-ALF.[9]

Liver transplant (LT) in ALF presents substantial challenges because of the rapidity and severity of illness (*i.e*. high risk of cerebral edema and multiorgan failure), the potential for recovery without LT, and the presence of concomitant complex psychosocial issues (contraindications) in several patients.[10–12] Given the advances in critical care management of ALF, some patients may have a good outcome despite meeting KCC and potentially avoid unnecessary LT in the presence of improved prognostic markers or scores.[7,13] There is an unmet need to discover and incorporate novel biomarkers of liver injury for prognostication of ALF and to determine which patients will require transplantation.

Inflammation and hepatocyte death underpin liver failure. Historically, necrosis and apoptosis have been considered the dominant modes of cell death in ALF;[14] however, more recently, programmed forms of pro-inflammatory cell death have been described. Necroptosis is a form of programmed necrosis that has a similar pathway and mediators of activation as apoptosis (*i.e*., tumor necrosis factor receptor 1 [TNFR1], TNFR2, TNF-related apoptosis including ligand [TRAIL] 1-2, and Fas).[15] However, under conditions of caspase-8 inhibition

* Corresponding author. Address: Department of Critical Care Medicine, Faculty of Medicine and Dentistry, College of Health Sciences and School of Public Health, University of Alberta, 2-139 Clinical Sciences Building, Edmonton, Alberta T6G-2B7, Canada. Tel.: +1 780 248 1555; fax: +1 780 492 5643.
E-mail address: dean.karvellas@ualberta.ca (C.J. Karvellas).
† Joint first authors.
https://doi.org/10.1016/j.jhepr.2026.101962

or ATP deficiency, this pathway leads to a lytic, pro-inflammatory cell death which morphologically resembles necrosis. The receptor-interacting protein kinases (RIPK) 1 and 3 are central to the necroptosis pathway through formation of the multimolecular necrosome complex, leading to phosphorylation of the effector protein mixed lineage kinase like protein (MLKL), which, in turn, forms pores within the cell membrane causing pro-inflammatory cell lysis. Importantly, necroptosis-independent and inflammation-dependent roles have been described for RIPK1, RIPK3, and MLKL.[16] Although the contribution of necroptosis in pre-clinical models of APAP-induced liver injury has been controversial, the potential for RIPKs and MLKL to act as prognostic biomarkers of inflammation and cell death in ALF has not been studied in humans.[17–23]

Pyroptosis is a further, pro-inflammatory mode of cell death implicated in liver disease. In a similar fashion, pyroptosis is characterized by lytic cell death following activation and cleavage of the effector proteins, gasdermins. The best described pathway is through activation of caspase(s)-1/4/5, leading to cleavage of gasdermin D (GSDMD), which then oligomerizes and integrates within the plasma membrane.[24] Gasdermin E (GSDME) is structurally similar, but it is cleaved by caspase-3, and therefore provides a link between apoptosis and pyroptosis.[25] Both the forms of pyroptosis lead to lytic cell death and release of pro-inflammatory damage-associated molecular patterns (DAMPs). Again, pre-clinical studies support a causal role for GSDMD and GSDME in liver disease, GSDMD in ALF,[26–29] and GSDME in steatohepatitis;[30] however, their prognostic role in patients with ALF remains unknown.

In this study, we aimed at determining the association of necroptosis and pyroptosis pathway proteins (RIPK3, MLKL, GSDMD, and GSDME) with clinical outcomes in a well-characterized ALF patient cohort. We tested the hypotheses that (1) lower serum levels of these biomarkers are associated with 21-day survival in the absence of LT, and (2) the addition of these biomarkers improves the performance of currently used ALF prognostic models including KCC, model for end-stage liver disease (MELD), and the ALFSG index.

Patients and methods

Study design and participants

This was a case-control study nested within the ALFSG registry. The ALFSG registry consists of 2,631 adult patients with ALF admitted to one of the 32 enrolling clinical sites from 1998 to 2019. Eligible patients with ALF had both coagulopathy (international normalized ratio [INR] ≥1.5) and any grade of HE (as clinically defined by the classic West Haven criteria) within 26 weeks of the onset of symptoms, and had no evidence of chronic liver disease.[31] All participating sites adhered to local institutional review board requirements, and written informed consent was obtained from the patient's legal next of kin.

Consistent with ALFSG studies,[31] the primary outcome of the current analysis was 21-day transplant-free survival (TFS). A total of 1,326 patients in the ALFSG registry were alive at Day 21 in the absence of LT, of whom 86 were randomly selected for the present analysis. Among the 1,303 patients who had non-TFS status, 90 were randomly selected (Fig. S1). Random selection was performed by personnel not involved in sample processing or statistical analysis for the paper, blinded to the clinical, biochemical, and demographic information of the patients other than the 21-day TFS status. A further eight healthy controls were analyzed for RIPK3, MLKL, GSDMD, and GSDME only.

Reporting of the analysis of this study followed the STROBE guidelines for reporting case-control studies.[32]

Clinical variables

Demographic data (age, race, and sex) were collected alongside biochemical parameters, which were prospectively measured on Day 1 and Day 4 and included complete blood count, INR, transaminases, bilirubin, ammonia, creatinine, lactate, and phosphate. The need for organ support, such as mechanical ventilation (MV), vasopressors, and renal replacement therapy (RRT), was documented as a binary variable (yes/no) from Day 1 to Day 7.

Prognostic scores were calculated on Day 1 and Day 4. The KCC predicts poor outcome (death/LT) in APAP-ALF if: (1) pH <7.3, or (2) INR >6.5, creatinine >3.4 mg/dl, and high HE coma grade (grade 3 or 4). In non-APAP drug-induced liver injury (DILI), KCC is defined as: (1) INR >6.5, or (2) any three of the following criteria: age <10 or >40 years, duration of jaundice before HE >7 days, INR >3.5, and serum bilirubin >300 μmol/L.[4] MELD is defined as:

$$10 \times (0.957 \times \log[4] + 0.378 \times \log[\text{bilirubin}] + 1.12 \times \log[\text{INR}]) \quad (1)$$

for dialysis patients and

$$10 \times (0.957 \times \log[\text{creatinine}] + 0.378 \times \log[\text{bilirubin}] + 1.12 * \log[\text{INR}]) \quad (2)$$

for non-dialysis patients.[33] The ALFSG index calculates the log odds (logit) for 21-day TFS as

$$2.67 - 0.95 \times (\text{HE}) + 1.56 \times (\text{etiology}) - 1.25 \times (\text{vasopressor}) - 0.70 \times \ln(\text{bilirubin}) - 1.35 \times \ln(\text{INR}).^{9} \quad (3)$$

HE = 1 if the coma grade is high (grade 3 or 4), etiology = 1 if APAP, and vasopressor use = 1 if present; otherwise, all are 0.

Laboratory assays of RIPK3, MLKL, GSDMD, and GSDME

The following ELISAs were performed in duplicate according to manufacturer's instructions: RIPK3 (Biorbyt, Durham, NC, USA; orb552189), MLKL (Biorbyt; orb443049), GSDMD (Abcam, Cambridge, UK; AB272463), and GSDME (Abbexa, Cambridge, UK; abx386859). Both Days 1 and 4 serum samples were analyzed in these assays.

Statistical analysis

Differences between TFS and non-TFS status among patients with ALF were compared using the Chi-square test for categorical variables. Continuous variables were reported as medians with IQR and compared using the Wilcoxon rank-sum test. Logistic regression analysis was performed to examine the association of RIPK3, MLKL, GSDMD, and GSDME with 21-day TFS. Biomarker levels are reported as absolute values and, in sensitivity analyses, as multiples of the upper limit of

normal (× ULN) observed in healthy controls. Covariates considered were etiology, MELD, vasopressor use, need for RRT, and MV; variance inflation factor (VIF) indicated no significant multicollinearity (VIF ≤1.74 for all). Separate models were derived for biomarkers and covariates measured on Day 1 and Day 4, as well as for changes (Δ = Day 4 − Day 1) in biomarkers, using complete-case analyses. To explore patient heterogeneity, we applied factor analysis of mixed data (FAMD) to admission clinical variables for all ALFSG patients with ALF with available baseline data (n = 2,619), without incorporating biomarker levels or TFS outcomes. The resulting dimensions were used for k-means clustering to identify potential patient subgroups, and associations between biomarkers and 21-day TFS were subsequently evaluated within each cluster. Sensitivity analysis was performed using Gaussian mixture model (GMM) clustering to address uncertainty in cluster assignment.

Receiver operating characteristic (ROC) curve analysis was conducted to assess the significance of adding RIPK3, MLKL, and GSDME to existing prognostic scores (KCC, MELD, and ALFSG index). An additional model using Day 1 measurements of RIPK3 and covariates was derived by performing backward elimination with a *p* value threshold of 0.05. Comparisons of the area under the ROC curve (AUROC) statistics between models (*e.g.* KCC vs. KCC + RIPK3) were performed using the DeLong method.[34] A 10-fold cross-validation was carried out to evaluate model generalizability, with AUROC calculated for each fold and averaged across all folds. The 95% CI for the mean AUROC was derived using the bootstrap method. R version 4.4.2 (R Foundation for Statistical Computing, Vienna, Austria) and SAS version 9.4 (SAS Institute, Cary, NC, USA) were used for the analysis. A two-sided *p* value of <0.05 was considered statistically significant.

Results

Patient characteristics

Of the 176 patients with ALF, 153 (87%) were diagnosed with APAP-ALF, whereas 23 (13%) developed ALF due to DILI. Furthermore, 86 patients spontaneously survived by Day 21 (TFS), 89 died, and one underwent LT. The most common causes of death were multiorgan failure (n = 43, 48%) and

Table 1. Patient characteristics by 21-day TFS status.

	Spontaneous alive at Day 21 (TFS, n = 86)	Dead/LT at or before Day 21 (non-TFS, n = 90)	*p* values
Age (years)	39 (29–50)	41 (31–56)	0.222
Etiology			
APAP	75 (87.2)	78 (86.7)	0.915
DILI	11 (12.8)	12 (13.3)	
Male	21 (24.4)	29 (32.2)	0.251
Race			
White/Caucasian	74 (86.0)	66 (73.3)	0.090
African American	6 (7.0)	15 (16.7)	
Other	6 (7.0)	9 (10.0)	
Organ support (Days 1–7)			
Mechanical ventilation	43 (50.0)	82 (91.1)	<0.001
Vasopressors use	24 (27.9)	66 (73.3)	<0.001
Renal replacement therapy	29 (33.7)	54 (60.0)	<0.001
KCC (Day 1)	10 (11.6)	23 (25.6)	0.018
HE coma grade 3 or 4* (Days 1–7)	45 (53.6)	79 (88.8)	<0.001
ICP-directed therapies (Days 1–7)			
ICP monitor	2 (2.3)	22 (24.4)	<0.001
Mannitol	7 (8.1)	32 (35.6)	<0.001
Hypertonic saline	11 (12.8)	14 (15.6)	0.599
Barbiturates	6 (7.0)	17 (18.9)	0.019
Hypothermia	3 (3.5)	15 (16.7)	0.004
Sedatives	44 (51.2)	78 (86.7)	<0.001
Need for blood products (Days 1–7)			
Red blood cells	23 (26.7)	47 (52.2)	<0.001
Fresh frozen plasma	26 (30.2)	69 (76.7)	<0.001
Recombinant VIIA	0 (0.0)	5 (5.6)	0.027
Platelets	13 (15.1)	36 (40.0)	<0.001
ICU complications (Days 1–7)			
Seizures	0 (0.0)	15 (16.7)	<0.001
Arrhythmias	14 (16.3)	33 (36.7)	0.002
Gastrointestinal bleeding	4 (4.7)	17 (18.9)	0.004
Abnormal chest X-ray	40 (46.5)	25 (27.8)	0.010
Infection (Days 1–7)			
Positive urinary tract infection	22 (25.6)	17 (18.9)	0.285
Positive tracheal aspirate	19 (22.1)	26 (28.9)	0.302
Bacteremia/blood stream infection	17 (19.8)	15 (16.7)	0.594
Any antibiotics	42 (48.8)	36 (40.0)	0.238
Prophylactic antibiotics	32 (37.2)	50 (55.6)	0.015
Listed for LT	14 (16.3)	28 (31.1)	0.021

Data are presented as median (IQR) or n (% of available data). *Missing data on hepatic encephalopathy (HE) grade (according to West Haven criteria) during the first 7 days of study admission: TFS group, n = 2; non-TFS group, n = 1. Mann-Whitney *U* test for continuous variables, Chi-square test for categorical variables. ALF, acute liver failure; APAP, acetaminophen, DILI, drug-induced liver injury; HE, hepatic encephalopathy; ICP, intracranial pressure; KCC, King's College Criteria; LT, liver transplant; TFS, transplant-free survival.

cerebral edema (n = 22, 25%); the cause of death was unknown in 24 (27%) patients.

Patient characteristics stratified by 21-day TFS status are shown in Table 1. There were no significant differences in age or sex between TFS and non-TFS. During the first 7 days of study admission, patients with TFS status required significantly less organ support than patients with non-TFS status, including MV (50% *vs*. 91%), vasopressor use (28% *vs*. 73%), and RRT (34% *vs*. 60%; p ≤0.0005 for all). They were also less likely to achieve grade 3 or 4 HE (54% *vs*. 89%) or to receive mannitol for intracranial hypertension (8% *vs*. 36%; p <0.0001 for both). Compared with patients with non-TFS status, patients with TFS status experienced fewer complications, such as seizures (0% *vs*. 17%), arrhythmias (16% *vs*. 37%), and gastrointestinal bleeding (5% *vs*. 19%; p ≤0.004 for all). On admission (Day 1), 12% of TFS patients and 26% of patients with non-TFS status met KCC (p = 0.018).

Clinical parameters measured on day 1

Comparison of clinical and biochemical parameters at study admission (Day 1) is presented in Table 2, with normalized levels (xULN) of RIPK3, MLKL, GSDMD, and GSDME shown in Table S1. Patients with TFS status were less likely to be on organ support or to achieve grade 3 or 4 HE, and they had lower admission MELD scores than patients with non-TFS status. Serum INR and levels of bilirubin, creatinine, and lactate were lower in patients with TFS than in non-TFS status.

Patients with TFS status had significantly lower Day 1 levels of RIPK3 (6.79 *vs*. 10.29 ng/ml) and MLKL (36.23 *vs*. 45.40 ng/ml, p <0.001 for both) than patients with non-TFS status (Table 2 and Fig. S2). Levels of RIPK3 and MLKL were also significantly lower in healthy controls than in patients with ALF (Table S2). For Day 1 levels of GSDMD and GSDME, no significant differences were noted between healthy controls and patients with ALF, or between patients with and without TFS status.

Clinical parameters measured on day 4

Parameters measured on Day 4 (Table 3, Table S1, and Fig. S2) were similar to those observed earlier on Day 1. Patients with TFS status remained less likely to require organ support or to develop grade 3 or 4 HE, with lower MELD scores and reduced serum INR, bilirubin, creatinine, and lactate levels compared with patients with non-TFS status.

TFS group had lower Day 4 levels of MLKL (16.13 *vs*. 19.10 ng/ml, p <0.001), but no significant difference in RIPK3 levels (7.28 *vs*. 8.00 ng/ml, p = 0.947) compared with non-TFS group. GSDME levels were significantly lower in TFS than in non-TFS group (0.68 *vs*. 1.70 ng/ml, p <0.001), whereas GSDMD levels did not significantly differ.

RIPK3, MLKL, GSDMD, and GSDME in association with 21-day TFS

The associations of RIPK3, MLKL, GSDMD, and GSDME with 21-day TFS were analyzed separately at two time points: on Day 1 (admission) and Day 4, with results presented in Table 4 and Table S3.

Lower Day 1 levels of RIPK3 were significantly associated with 21-day TFS (odds ratio [OR] 1.09 per 1 ng/ml decrease, 95% CI 1.04–1.15). This association remained after adjusting

Table 2. Clinical and biochemical parameters measured on Day 1.

	Spontaneous alive at Day 21 (TFS, n = 86)		Dead/LT at or before Day 21 (non-TFS, n = 90)		
	N	n (%)/median (IQR)	N	n (%)/median (IQR)	*p* values
Organ support					
Mechanical ventilation	86	36 (41.9)	90	65 (72.2)	<0.001
Vasopressors use	86	16 (18.6)	90	38 (42.2)	<0.001
Renal replacement therapy	86	17 (19.8)	90	32 (35.6)	0.020
MELD	84	29 (23–32.5)	87	36 (29–42)	<0.001
HE coma grade 3 or 4*	83	36 (43.4)	88	57 (64.8)	0.005
Biochemistry					
Hemoglobin (g/dl)	86	10.8 (9.2–12.7)	90	10.6 (29.3–12.3)	0.885
White blood count (10^9/L)	85	8.8 (6.3–12.0)	90	11.9 (9.0–16.7)	<0.001
Neutrophils (10^9/L)	54	83 (73–90)	63	88.5 (81–94)	0.021
Lymphocytes (10^9/L)	59	9.0 (5.4–14.0)	69	7.0 (4.0–13.0)	0.206
Monocytes (10^9/L)	60	3.6 (1.0–6.9)	68	2.0 (1.0–5.5)	0.064
Platelet count (10^9/L)	85	132 (92–187)	90	119 (71–165)	0.176
INR	85	2.7 (1.9–3.8)	87	3.5 (2.5–4.9)	<0.001
ALT (IU/L)	86	3,666.5 (1,690–5,637)	90	2,744.5 (1,292–5,223)	0.166
Bilirubin (mg/dl)	85	4.4 (2.5–6.6)	90	5.6 (3.7–9.6)	<0.001
Ammonia (venous) (µmol/L)	45	89 (57–110)	31	122 (83–293)	0.005
Creatinine (mg/dl)	86	1.5 (0.82–2.9)	90	2.3 (1.2–3.8)	0.006
Lactate (mmol/L)	58	2.9 (1.9–4.7)	68	7.2 (4.0–11.1)	<0.001
Phosphate (mg/dl)	77	2.4 (1.7–3.3)	79	3.4 (2.3–4.8)	<0.001
RIPK3 (ng/ml)	84	6.79 (4.57–9.46)	89	10.29 (6.36–15.30)	<0.001
MLKL (ng/ml)	86	36.23 (29.80–46.60)	89	45.40 (36.33–51.07)	<0.001
GSDMD (ng/ml)	86	2.46 (1.46–3.04)	89	2.64 (1.75–3.97)	0.074
GSDME (ng/ml)	86	2.02 (0.85–3.84)	89	2.80 (1.15–4.72)	0.135

*According to West Haven criteria. Mann-Whitney *U* test for continuous variables, Chi-square test for categorical variables. ALT, alanine aminotransferase; GSDMD, gasdermin D; GSDME, gasdermin E; HE, hepatic encephalopathy; INR, international normalized ratio; LT, liver transplant; MELD, model for end-stage liver disease; MLKL, mixed lineage kinase domain-like protein; RIPK3, receptor-interacting protein kinase 3; TFS, transplant-free survival.

Table 3. Clinical and biochemical parameters measured on Day 4.

	Spontaneous alive at Day 21 (TFS, n = 86)		Dead/LT at or before Day 21 (non-TFS, n = 90)		
	N	n (%)/median (IQR)	N	n (%)/median (IQR)	*p* value
Organ support					
Mechanical ventilation	86	37 (43.0)	90	82 (91.1)	<0.001
Vasopressors	86	15 (17.4)	90	53 (58.9)	<0.001
Renal replacement therapy	86	22 (25.6)	90	46 (51.1)	<0.001
MELD	78	22 (17–28)	56	32 (25-40)	<0.001
HE coma grade 3 or 4*	65	33 (50.8)	86	76 (88.4)	<0.001
Biochemistry					
Hemoglobin (g/dl)	82	10.1 (8.8–11.0)	62	9.7 (8.6–10.9)	0.662
White blood count (10^9/L)	81	S8.6 (6.2–12.8)	62	10.9 (7.0–15.0)	0.107
Neutrophils (10^9/L)	24	70.6 (60.5–78.8)	17	78 (73–84)	0.078
Lymphocytes (10^9/L)	26	14.1 (10.0–21.4)	18	8.3 (4.0–16.0)	0.012
Monocytes (10^9/L)	26	10.5 (6.3–14.5)	18	7.0 (2.9–13.0)	0.181
Platelet count (10^9/L)	82	97.5 (59–161)	62	70.5 (44–109)	0.006
INR	82	1.55 (1.30–2.00)	59	2.3 (1.7–3.4)	<0.001
ALT (IU/L)	83	1,207 (718–1,960)	60	775 (362.5–1,730.5)	0.063
Bilirubin (mg/dl)	82	6.1 (2.7–10.1)	60	12.3 (7.8–16.3)	<0.001
Ammonia (venous) (μmol/L)	22	57 (45–75)	15	95 (64–133)	0.013
Creatinine (mg/dl)	83	1.04 (0.6–2.1)	60	1.75 (1.24–3.11)	<0.001
Lactate (mmol/L)	19	1.9 (1.3–2.9)	29	3.2 (2.5–5.0)	<0.001
Phosphate (mg/dl)	54	2.7 (2.3–3.4)	30	3.3 (2.6–4.1)	0.053
RIPK3 (ng/ml)	85	7.28 (4.44–11.33)	53	8.00 (3.83–12.83)	0.947
MLKL (ng/ml)	85	16.13 (13.36–17.78)	53	19.10 (16.92–20.47)	<0.001
GSDMD (ng/ml)	85	2.40 (1.68–4.57)	53	2.17 (1.49–4.39)	0.676
GSDME (ng/ml)	84	0.67 (0.29–1.38)	53	1.70 (0.76–2.47)	<0.001

*According to West Haven criteria. Mann-Whitney *U* test for continuous variables, Chi-square test for categorical variables. ALT, alanine aminotransferase; GSDMD, gasdermin D; GSDME, gasdermin E; HE, hepatic encephalopathy; INR, international normalized ratio; LT, liver transplant; MELD, model for end-stage liver disease; MLKL, mixed lineage kinase domain-like protein; RIPK3, receptor-interacting protein kinase 3; TFS, transplant-free survival.

for RRT and other clinically significant covariates (OR 1.10 per 1 ng/ml decrease, 95% CI 1.03–1.17) and persisted in patients off RRT with further adjustment for serum creatinine (OR 1.08 per 1 ng/ml decrease, 95% CI 1.01–1.16, p = 0.028; n = 123), consistent with an effect beyond impaired renal clearance. Lower Day 1 MLKL levels were associated with 21-day TFS in the unadjusted analysis but were non-significant after covariate adjustment. No significant associations were observed between Day 1 levels of GSDMD or GSDME and 21-day TFS.

In unadjusted analyses, lower Day 4 levels of MLKL and GSDME were associated with 21-day TFS. However, these associations were not observed after adjusting for clinically significant covariates. Day 4 RIPK3 and GSDMD levels showed no significant association with 21-day TFS. Adjusted ORs for all biomarkers on Day 1 and Day 4 were largely unchanged in bootstrap resampling (1,000 iterations; Table S4), confirming the stability of effect estimates.

FAMD applied to admission clinical variables for all ALFSG patients with ALF explained 48.2% of the total variance across five dimensions (Table S5), highlighting substantial patient heterogeneity. K-means clustering based on these dimensions identified two subgroups (Table S6): cluster 1 showed lower use of organ support, lower coma grades, and milder laboratory abnormalities, whereas cluster 2 had higher levels across these variables, reflecting more severe physiological derangement at admission. Cluster-specific analyses showed that Day 1 RIPK3 was significantly associated with 21-day TFS in cluster 2 (adjusted OR 1.13 per 1 ng/ml decrease, 95% CI

Table 4. Day 1 and Day 4 levels of RIPK3, MLKL, GSDMD, and GSDME in association with 21-day TFS.

	Unadjusted			Adjusted*		
	TFS/N	OR (95% CI)†	*p* values	TFS/N	OR (95% CI)†	*p* values
Day 1						
RIPK3	84/173	1.09 (1.04–1.15)	0.001	82/168	1.10 (1.03–1.17)	0.004
MLKL	86/175	1.05 (1.02–1.08)	0.001	84/170	1.01 (0.98–1.05)	0.509
GSDMD	86/175	1.14 (0.96–1.34)	0.133	84/170	1.19 (0.98–1.44)	0.077
GSDME	86/175	1.08 (0.96–1.20)	0.189	84/170	0.99 (0.86–1.15)	0.895
Day 4						
RIPK3	85/138	1.02 (0.98–1.05)	0.426	77/123	0.99 (0.93–1.06)	0.787
MLKL	85/138	1.42 (1.22–1.64)	<0.001	77/123	1.14 (0.93–1.41)	0.202
GSDMD	85/138	1.00 (0.96–1.04)	0.941	77/123	0.98 (0.92–1.05)	0.633
GSDME	84/137	1.32 (1.09–1.60)	0.005	77/123	1.00 (0.88–1.14)	0.983

‡Univariable and multivariable logistic regression, boot strapping, Wald test). APAP, acetaminophen, DILI, drug-induced liver injury; GSDMD, gasdermin D; GSDME, gasdermin E; MLKL, mixed lineage kinase domain-like protein; OR, odds ratio; RIPK3, receptor-interacting protein kinase 3; TFS, transplant-free survival.

*Adjusted for etiology (APAP or DILI), MELD, vasopressors use, need for renal replacement therapy and mechanical ventilation, all recorded on the same day as the biomarker measurement (Day 1 or Day 4); degrees of freedom = 6.

†Per 1 ng/ml decrease.

1.03–1.27), whereas no significant associations were found for cluster 1 or for other biomarkers (Table S7). GMM clustering using FAMD dimensions yielded consistent results (Tables S8 and S9), with low classification uncertainty; only 4% of patients had a maximum posterior probability below 0.7. Together, these findings suggest that the prognostic relevance of RIPK3 is more pronounced in patients with greater clinical severity.

Performance of day 1 RIPK3 and MLKL in predicting TFS

Given the significant associations between Day 1 levels of RIPK3 and MLKL and 21-day TFS, we further explored the incremental benefit of adding RIPK3 and MLKL to established clinical models (KCC, MELD, and the ALFSG index) for predicting TFS.

When assessed at admission (Day 1), the AUROC values were 0.57 (95% CI 0.51–0.63) for KCC, 0.72 (95% CI 0.64–0.80) for MELD, and 0.80 (95% CI 0.73–0.87) for the ALFSG index. As shown in Fig. 1 and Table S10, adding RIPK3 and MLKL to KCC improved the AUROC to 0.67 (95% CI 0.59–0.75) and 0.70 (95% CI 0.62–0.78), respectively, significantly outperforming KCC alone (p ≤0.003 for both). No significant improvement was observed when RIPK3 or MLKL were added to MELD or ALFSG index.

We conducted a backward selection of covariates listed in Table 2, along with RIPK3 (all measured on Day 1), in association with 21-day TFS. The final model, comprising RIPK3 (OR 1.09 per 1 ng/ml decrease, 95% CI 1.03–1.16), MELD (OR 1.09 per 1 point decrease, 95% CI 1.04–1.15), the use of vasopressors (OR 0.33, 95% CI 0.14–0.77), and MV (OR 0.15, 95% CI 0.05–0.43), achieved an AUROC of 0.87 (95% CI 0.81–0.92), significantly outperforming the ALFSG index (p = 0.012; Fig. 1A). In 10-fold cross-validation, the model's mean AUROC was 0.85 (95% CI 0.77–0.89), indicating good discriminative ability and generalizability (Table S11).

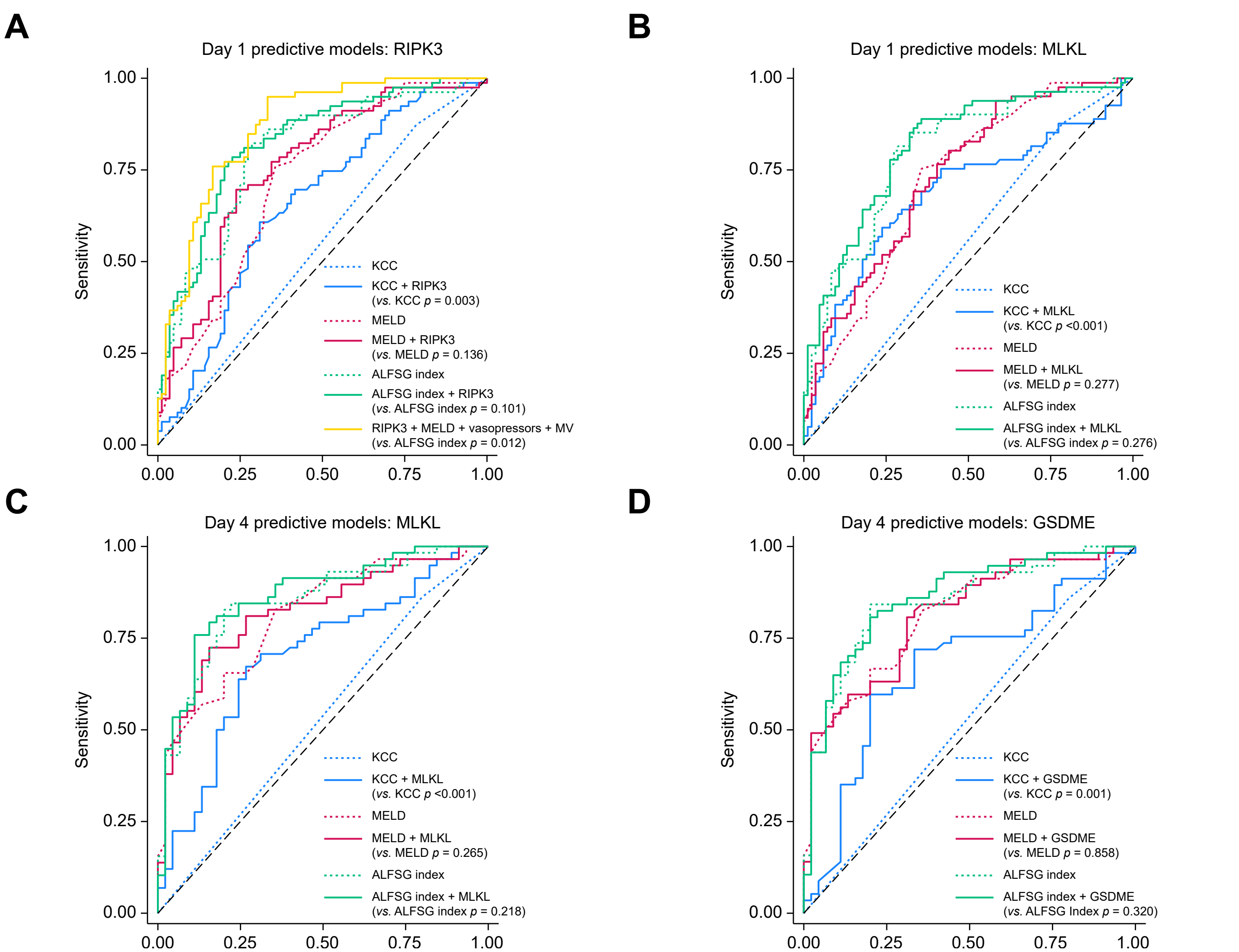

Fig. 1. ROC curves for predictive models. ROC curves for predictive models assessed on Day 1 with and without (A) RIPK3 and (B) MLKL, and assessed on Day 4 with and without (C) MLKL and (D) GSDME. The p values are derived from pairwise comparison of ROC curves using DeLong tests. AUROC values and 95% CIs are in Table S10. ALFSG, Acute Liver Failure Study Group; AUROC, area under the ROC curve; GSDME, gasdermin E; KCC, King's College Criteria; MELD, model for end-stage liver disease; MLKL, mixed lineage kinase domain-like protein; RIPK3, receptor-interacting protein kinase 3; ROC, receiver operator curve.

Performance of day 4 MLKL and GSDME in predicting TFS

When measured on Day 4, the AUROC values were 0.57 (95% CI 0.51–0.63) for KCC, 0.82 (95% CI 0.74–0.89) for MELD, and 0.84 (95% CI 0.77–0.91) for ALFSG index. Day 4 levels of MLKL and GSDME were associated with 21-day TFS (Table 4) and, in our further analysis, led to a significant improvement in AUROC when added to KCC, compared with KCC alone (Fig. 1 and Table S10). However, adding MLKL and GSDME did not result in any significant improvement over MELD or ALFSG index alone.

The performance of all predictive models, assessed on Day 1 or Day 4, remained consistent during cross-validation, with the mean AUROC values for each model presented in Table S11.

Secondary analysis: temporal changes in RIPK3, MLKL, GSDMD, and GSDME and 21-day TFS

As a secondary mechanistic analysis (Table S12), we examined whether changes in RIPK3, MLKL, GSDMD, and GSDME between Day 1 and Day 4 (Δ = Day 4 – Day 1) were associated with 21-day TFS. In models adjusted for Day 1 levels, greater reductions in MLKL (adjusted OR 1.47 per 1 ng/ml larger decrease, 95% CI 1.23–1.75, p <0.001) and GSDME (OR 1.30, 95% CI 1.07–1.59, p = 0.010) were associated with higher TFS, whereas changes in RIPK3 and GSDMD were not significant. After further adjustment for clinical covariates, the association remained significant for MLKL (adjusted OR 1.24, 95% CI 1.02–1.52, p = 0.035) and borderline for GSDME (OR 1.26, 95% CI 0.99–1.62, p = 0.063).

Discussion

Key results

This study, as part of the ALFSG, reports a model for predicting 21-day survival without LT using the novel, discovery-phase biomarker RIPK3. Importantly, although the contribution of cell death pathway proteins (necroptosis and pyroptosis) has been extensively studied in pre-clinical models, these are the first data examining these key proteins in patients with ALF. These findings are of great importance in view of the need for accurate prognostic tools to guide use of LT, and the potential to develop novel therapeutic strategies.

The principal findings of this nested case-control study are: (1) an independent association between lower serum RIPK3 levels at the time of admission and 21-day survival in the absence of LT, (2) a novel predictive model of serum RIPK3 that outperforms the ALFSG index, and (3) improved performance of KCC following the addition of RIPK3, MLKL, or GSDME.

Strengths and limitations

This study represents the first systematic evaluation of inflammatory cell death markers in a large, multicenter, ALF patient cohort. The ALFSG registry represents the largest prospectively collected patient cohort for the study of this rare disease, with detailed and prospectively maintained clinical and laboratory data. As such, this nested case-control design represents a unique opportunity to study novel cell death markers for mechanistic and biomarker purposes. Additionally, samples were collected at two time points, allowing for a dynamic evaluation of biomarkers, along with comparisons to samples from healthy controls.

There are several limitations to this study that warrant consideration. First, as this is a nested case-control study, the event rate of 21-day TFS is different from that published in other cohort series. Because the true baseline risk is not preserved, our analysis can inform association and discrimination (between TFS and non-TFS) but not absolute risk estimates. The case-control design may also have introduced selection bias because the primary outcome of TFS is automatically unbalanced within the clinical profile of the groups. In an attempt to reduce bias, data was collected prospectively, and, within this specific study design, researchers measuring RIPK3, MLKL, GSDMD, and GSDME were blinded to all clinical and outcome data of the patients. Cross-validation assessed internal model performance. FAMD-based clustering demonstrated that the association between admission RIPK3 levels and TFS was preserved and strongest in patients with greater clinical severity; nevertheless, whether this reflects effect modification by severity or a distinct biological phenotype remains unclear and should be interpreted cautiously. Second, although we adjusted for a range of clinically significant covariates, residual confounding could not be excluded. Admission lactate data were unavailable for 50 patients, and further adjustment for lactate in the multivariable analysis yielded a borderline significant OR of 1.09 (95% CI 1.00–1.17) for RIPK3 in association with 21-day TFS, likely because of the reduced sample size of 119. There may also be confounding due to inconsistencies in transplant listing and organ availability across sites; however, our results are unlikely to be affected, as only one patient in this study received LT. Third, KCC was treated as a dichotomized variable. Although this reflects its established use in clinical practice as a decision-making tool, it may reduce the predictive ability and lead to loss of information given its composite nature. The small number of healthy controls further limits the precision and interpretability of biomarker ULN. Finally, the cohort presented largely reflects APAP-related ALF; therefore, we must be cautious in generalizing these findings to non-APAP etiologies of ALF.

Comparison with literature

Programmed, non-apoptotic cell death pathways are increasingly recognized to play a crucial role in host response to pathogens and sterile inflammation. Biomarkers for several non-apoptotic pathways (*e.g.* necroptosis, pyroptosis, and ferroptosis) have been found to be elevated in critical illness and associated with organ dysfunction. The necroptosis marker RIPK3 is reportedly elevated in critically ill patients with sepsis and associated with higher mortality.[35] Similarly, the pyroptosis marker GSDMD was elevated in critically ill patients with acute respiratory distress syndrome.[36] From a mechanistic perspective, necroptosis and pyroptosis are lytic, pro-inflammatory pathways that promote the release of intracellular DAMPs. Pre-clinical data suggest this lytic cell death plays an important role in mounting an immune response against infectious insults, particular intracellular pathogens.[36] However, these pathways are increasingly thought to contribute to aberrant tissue injury and adverse outcomes in

sepsis and chronic inflammatory disease, although causal evidence is limited to pre-clinical models.

In the context of liver disease, non-apoptotic cell death markers have been associated with both acute and chronic liver injury. In ALF, Kondo *et al.*[37] recently demonstrated an association of serum RIPK3 levels with mortality in a cohort of 71 patients with acute liver injury, including 11 with ALF; no variables were found to significantly predict survival on multivariable analysis, likely because of the small size of the cohort. From a mechanistic perspective the situation is more complex, and there has been controversy surrounding the role of the RIPKs (RIPK1 and RIPK3) and the occurrence of necroptosis in pre-clinical APAP-ALF models.[17,22,38] Hepatocytes do not express RIPK3 at baseline, although immune cells and liver endothelial cells express abundant amounts of RIPK3; it is therefore likely that elevated circulating levels of RIPK3 in ALF reflect systemic inflammatory injury and regulated necrosis rather than hepatocyte-specific necroptosis alone.[39–41] Further work is required to fully delineate the cellular origin of RIPK3 in acute liver injury.

Phosphorylated MLKL (p-MLKL) is the effector protein of necroptotic cell death, causing membrane pore formation and lytic cell death.[42] In this study, we measured total MLKL rather than p-MLKL. Nevertheless, MLKL levels were consistently lower in TFS than in non-TFS group, likely a reflection of MLKL being downstream in the necroptosis pathway. Greater decline in MLKL levels between Day 1 and Day 3 was also associated with TFS, possibly reflecting the resolution of necroptosis and recovery. However, we acknowledge that although these associations are intriguing, we cannot make firm mechanistic conclusions regarding the role of necroptosis in the absence of p-MLKL measurement.

The data presented here do not support an association between GSDMD or GSDME levels and outcome in ALF. This suggests the pyroptosis pathway is not a major effector of systemic inflammation in acute liver injury. This is likely a different situation to patients with established cirrhosis and superimposed multiorgan failure (acute-on-chronic liver failure [ACLF]), where chronic injury, immune priming, and the altered hepatic microenvironment may sensitize lytic cell death pathways. Consistent with this, our own data and that of others demonstrate upregulation of the hepatocyte caspase-4/GSDMD pathway in fibrosis and steatohepatitis, thus supporting increased susceptibility to pyroptosis in chronic liver disease. Pre-clinical models further support a causal role for GSDMD in the development of ACLF.[43,44] Nevertheless, the situation in ACLF is likely more complex and may depend on etiology and precipitating event. Recently, Verma *et al.*[45] presented a biomarker study exploring RIPK3 and GSDMD in patients with decompensated cirrhosis or ACLF, predominantly with alcohol-related liver disease. This group found elevations of RIPK3 and GSDMD, although RIPK3 levels provided the strongest prognostic discrimination. Conversely, Zhao *et al.*[46] recently provided mechanistic evidence of GSDMD-mediated pyroptosis in hepatitis B-related ACLF. This group used unbiased multi-omic methods to demonstrate that pyroptosis is the dominant liver cell death pathway, and *ex vivo* liver function could be improved with antipyroptosis therapy.

From a translational perspective, there is currently no Good Laboratory Practice-validated assay for RIPK3, and therefore integration into transplantation decision-making pathways is not yet feasible. Therefore, RIPK3 remains a discovery-phase biomarker, although the results presented here support the development of an assay suitable for regulatory approval, and prospective studies of RIPK3 as a clinical ALF biomarker.

Conclusions

Serum levels of the necroptosis mediator RIPK3 are associated with TFS in ALF. GSDMD and GSDME levels were not associated with outcome in ALF. These data support validation studies of RIPK3 as a mechanistic biomarker in ALF, and further translational work of RIPK3 inhibition as a potential treatment modality for ALF.

Affiliations

[1]*Institute for Liver and Digestive Health, University College London, London, UK;* [2]*Roger Williams Institute of Hepatology, Foundation for Liver Research, London, UK;* [3]*Department of Biostatistics and Data Science, Division of Public Health Sciences, Wake Forest University School of Medicine, Winston-Salem, NC, USA;* [4]*Department of Medicine, University of Southern California, Los Angeles, CA, USA;* [5]*Department of Public Health Sciences, Medical University of South Carolina, Charleston, SC, USA;* [6]*Division of Digestive and Liver Diseases, Department of Internal Medicine, University of Texas Southwestern Medical Center, Dallas, TX, USA;* [7]*Divisions of Hepatology and Critical Care Medicine, University of Alberta, Edmonton, Canada*

Abbreviations

ACLF, acute-on-chronic liver failure; ALF, acute liver failure; ALFSG, Acute Liver Failure Study Group; ALT, alanine aminotransferase; APAP, acetaminophen; DAMPs, damage-associated molecular patterns; DILI, drug-induced liver injury; FAMD, factor analysis of mixed data; GMM, Gaussian mixture model; GSDMD, gasdermin D; GSDME, gasdermin E; HE, hepatic encephalopathy; ICP, intracranial pressure; INR, international normalized ratio; KCC, King's College Criteria; LT, liver transplantation; MELD, model for end-stage liver disease; MLKL, mixed lineage kinase domain-like; MV, mechanical ventilation; OR, odds ratio; p-MLKL, phosphorylated MLKL; RIPK3, receptor-interacting protein kinase 3; ROC, receiver operator curve; RRT, renal replacement therapy; TFS, transplant-free survival; TNF, tumor necrosis factor; ULN, upper limit of normal; VIF, variance inflation factor.

Financial support

The study was sponsored by National Institutes of Health grant U-01 58369 from the National Institute of Diabetes, Digestive and Kidney Diseases.

Conflicts of interest

GM has shares in Hepyx Ltd and Yaqrit Ltd. CJK reports receiving consulting fees from Genfit, Vantive, Mallinckrodt Pharmaceuticals, Morphocell Inc., and DSMB committee service for Miromatrix. All other authors have no personal or funding conflicts of interest. Please refer to the accompanying ICMJE disclosure forms for further details.

Authors' contributions

Study conception and design: GM, CJK. Laboratory analysis: US. Data analysis: GM, CJK. Data interpretation: GM, CD, JLS, CJK. Statistical analysis: CD, JLS, VD. Dataset preparation: SB, VD. Drafted the final manuscript: GM, CJK. Critical revision of the final manuscript: CD, JLS, SB, LD, VD, WML. Supervision of entire US Acute Liver Failure Study Group (U-01 Grant): WML.

Data availability

Data presented in this study are available on application to the ALFSG.

Acknowledgments

Members and institutions participating in the Acute Liver Failure Study Group 1998–2018 are as follows: William M. Lee (Principal investigator); Anne M. Larson and Iris Liou (University of Washington, Seattle, WA, USA); Oren Fix (Swedish Medical Center, Seattle, WA, USA); Michael Schilsky (Yale University, New Haven, CT, USA); Timothy McCashland (University of Nebraska, Omaha, NE, USA); J. Eileen Hay (Mayo Clinic, Rochester, MN, USA); Natalie Murray (Baylor University Medical Center, Dallas, TX, USA); A. Obaid S. Shaikh (University of Pittsburgh, Pittsburgh, PA, USA); Andres Blei (deceased; Northwestern University, Chicago, IL, USA); Daniel Ganger (Northwestern University, Chicago, IL, USA); Atif Zaman (University of Oregon, Portland, OR, USA); Steven H.B. Han (University of California, Los Angeles, CA, USA); Robert Fontana (University of Michigan, Ann Arbor, MI, USA); Brendan McGuire (University of Alabama, Birmingham, AL, USA); Raymond T. Chung (Massachusetts General Hospital, Boston, MA, USA); Alastair Smith (Duke University Medical Center, Durham, NC, USA); Robert Brown (Cornell/Columbia University, New York, NY, USA); Jeffrey Crippin (Washington University, St Louis, MO, USA); Edwin Harrison (Mayo Clinic, Scottsdale, AZ, USA); Adrian Reuben (Medical University of South Carolina, Charleston, SC, USA); Santiago Munoz (Albert Einstein Medical Center, Philadelphia, PA); Rajender Reddy (University of Pennsylvania, Philadelphia, PA, USA); R. Todd Stravitz (Virginia Commonwealth University, Richmond, VA, USA); Lorenzo Rossaro (University of California Davis, Sacramento, CA, USA); Raj Satyanarayana (Mayo Clinic, Jacksonville, FL, USA); Tarek Hassanein (University of California, San Diego, CA, USA); Constantine J. Karvellas (University of Alberta, Edmonton, AB, USA); Jodi Olson (University of Kansas, Kansas City, KA, USA); Ram Subramanian (Emory, Atlanta, GA, USA); James Hanje (Ohio State University, Columbus, OH, USA); Bilal Hameed (University of California San Francisco, CA, USA). The University of Texas Southwestern Administrative Group included Grace Samuel, Ezmina Lalani, Carla Pezzia, and Corron Sanders, Nahid Attar, Linda S. Hynan. The Medical University of South Carolina Data Coordination Unit included Valerie Durkalski, Wenle Zhao, Jaime Speiser, Catherine Dillon, Holly Battenhouse, and Michelle Gottfried. This paper followed the STROBE guideline for reporting cohort studies (BMJ 2007): see Supplementary File 2.

Supplementary data

Supplementary data to this article can be found online at https://doi.org/10.1016/j.jhepr.2026.101962.

References

[1] O'Grady JG, Williams R. Classification of acute liver failure. Lancet 1993;342:743.
[2] Fagan E, Wannan G. Reducing paracetamol overdoses. BMJ 1996;313:1417–1418.
[3] Larson AM, Polson J, Fontana RJ, et al. Acetaminophen-induced acute liver failure: results of a United States multicenter, prospective study. Hepatology 2005;42:1364–1372.
[4] O'Grady JG, Alexander GJ, Hayllar KM, et al. Early indicators of prognosis in fulminant hepatic failure. Gastroenterology 1989;97:439–445.
[5] Speiser JL, Lee WM, Karvellas CJ, et al. Predicting outcome on admission and post-admission for acetaminophen-induced acute liver failure using classification and regression tree models. PLoS One 2015;10:e0122929.
[6] Schmidt LE, Dalhoff K. Serum phosphate is an early predictor of outcome in severe acetaminophen-induced hepatotoxicity. Hepatology 2002;36:659–665.
[7] Schmidt LE, Larsen FS. MELD score as a predictor of liver failure and death in patients with acetaminophen-induced liver injury. Hepatology 2007;45:789–796.
[8] Bernal W, Donaldson N, Wyncoll D, et al. Blood lactate as an early predictor of outcome in paracetamol-induced acute liver failure: a cohort study. Lancet 2002;359:558–563.
[9] Koch DG, Tillman H, Durkalski V, et al. Development of a model to predict transplant-free survival of patients with acute liver failure. Clin Gastroenterol Hepatol 2016;14:1199. 206.e2.
[10] Karvellas CJ, Safinia N, Auzinger G, et al. Medical and psychiatric outcomes for patients transplanted for acetaminophen-induced acute liver failure: a case-control study. Liver Int 2010;30:826–833.
[11] Bernal W, Auzinger G, Dhawan A, et al. Acute liver failure. Lancet 2010;376:190–201.
[12] Reddy KR, Schilsky M, Stravitz RT, et al. Liver transplantation for acute liver failure: results from the NIH acute liver failure study group. Hepatology 2012;56:246A.
[13] Shakil AO, Kramer D, Mazariegos GV, et al. Acute liver failure: clinical features, outcome analysis, and applicability of prognostic criteria. Liver Transpl 2000;6:163–169.
[14] Schulze-Osthoff K, Bantel H. Necrosis versus apoptosis in acetaminophen-induced hepatotoxicity. Hepatology 2011;53:1070.
[15] Schwabe RF, Luedde T. Apoptosis and necroptosis in the liver: a matter of life and death. Nat Rev Gastroenterol Hepatol 2018;15:738–752.
[16] Moriwaki K, Chan FK. Necroptosis-independent signaling by the RIP kinases in inflammation. Cell Mol Life Sci 2016;73:2325–2334.
[17] An J, Mehrhof F, Harms C, et al. ARC is a novel therapeutic approach against acetaminophen-induced hepatocellular necrosis. J Hepatol 2013;58:297–305.
[18] Ramachandran A, McGill MR, Xie Y, et al. Receptor interacting protein kinase 3 is a critical early mediator of acetaminophen-induced hepatocyte necrosis in mice. Hepatology 2013;58:2099–2108.
[19] Suda J, Dara L, Yang L, et al. Knockdown of RIPK1 markedly exacerbates murine immune-mediated liver injury through massive apoptosis of hepatocytes, independent of necroptosis and inhibition of NF-kappaB. J Immunol 2016;197:3120–3129.
[20] Dara L, Johnson H, Suda J, et al. Receptor interacting protein kinase 1 mediates murine acetaminophen toxicity independent of the necrosome and not through necroptosis. Hepatology 2015;62:1847–1857.
[21] Gunther C, He GW, Kremer AE, et al. The pseudokinase MLKL mediates programmed hepatocellular necrosis independently of RIPK3 during hepatitis. J Clin Invest 2016;126:4346–4360.
[22] Zhou Y, Dai W, Lin C, et al. Protective effects of necrostatin-1 against concanavalin A-induced acute hepatic injury in mice. Mediators Inflamm 2013;2013:706156.
[23] Weinlich R, Oberst A, Dillon CP, et al. Protective roles for caspase-8 and cFLIP in adult homeostasis. Cell Rep 2013;5:340–348.
[24] Liu X, Zhang Z, Ruan J, et al. Inflammasome-activated gasdermin D causes pyroptosis by forming membrane pores. Nature 2016;535:153–158.
[25] Rogers C, Fernandes-Alnemri T, Mayes L, et al. Cleavage of DFNA5 by caspase-3 during apoptosis mediates progression to secondary necrotic/pyroptotic cell death. Nat Commun 2017;8:14128.
[26] Li H, Zhao XK, Cheng YJ, et al. Gasdermin D-mediated hepatocyte pyroptosis expands inflammatory responses that aggravate acute liver failure by upregulating monocyte chemotactic protein 1/CC chemokine receptor-2 to recruit macrophages. World J Gastroenterol 2019;25:6527–6540.
[27] Wu YL, Ou WJ, Zhong M, et al. Gasdermin D inhibitor necrosulfonamide alleviates lipopolysaccharide/D-galactosamine-induced acute liver failure in mice. J Clin Transl Hepatol 2022;10:1148–1154.
[28] Zhou XN, Zhang Q, Peng H, et al. Silent information regulator sirtuin 1 ameliorates acute liver failure via the p53/glutathione peroxidase 4/gasdermin D axis. World J Gastroenterol 2024;30:1588–1608.
[29] Yuan X, Chen P, Luan X, et al. NLRP3 deficiency protects against acetaminophen-induced liver injury by inhibiting hepatocyte pyroptosis. Mol Med Rep 2024;29:61.
[30] Zhu JH, Ouyang SX, Zhang GY, et al. GSDME promotes MASLD by regulating pyroptosis, Drp1 citrullination-dependent mitochondrial dynamic, and energy balance in intestine and liver. Cell Death Differ 2024;31:1467–1486.
[31] Reuben A, Tillman H, Fontana RJ, et al. Outcomes in adults with acute liver failure between 1998 and 2013: an observational cohort study. Ann Intern Med 2016;164:724–732.
[32] von Elm E, Altman DG, Egger M, et al. Strengthening the Reporting of Observational Studies in Epidemiology (STROBE) statement: guidelines for reporting observational studies. BMJ 2007;335:806–808.
[33] Kamath PS, Wiesner RH, Malinchoc M, et al. A model to predict survival in patients with end-stage liver disease. Hepatology 2001;33:464–470.
[34] DeLong ER, DeLong DM, Clarke-Pearson DL. Comparing the areas under two or more correlated receiver operating characteristic curves: a nonparametric approach. Biometrics 1988;44:837–845.
[35] Choi H, Yoo H, Lee JY, et al. Plasma mitochondrial DNA and necroptosis as prognostic indicators in critically ill patients with sepsis. Biomedicines 2022;10:2386.
[36] Mitra S, Exline M, Habyarimana F, et al. Microparticulate caspase 1 regulates gasdermin D and pulmonary vascular endothelial cell injury. Am J Respir Cell Mol Biol 2018;59:56–64.
[37] Kondo T, Fujimoto K, Fujiwara K, et al. Potential of circulating receptor-interacting protein kinase 3 levels as a marker of acute liver injury. Sci Rep 2023;13:14043.

[38] Deutsch M, Graffeo CS, Rokosh R, et al. Divergent effects of RIP1 or RIP3 blockade in murine models of acute liver injury. Cell Death Dis 2015;6:e1759.
[39] Orozco S, Oberst A. RIPK3 in cell death and inflammation: the good, the bad, and the ugly. Immunol Rev 2017;277:102–112.
[40] Newton K, Dugger DL, Maltzman A, et al. RIPK3 deficiency or catalytically inactive RIPK1 provides greater benefit than MLKL deficiency in mouse models of inflammation and tissue injury. Cell Death Differ 2016;23:1565–1576.
[41] Dara L. The receptor interacting protein kinases in the liver. Semin Liver Dis 2018;38:73–86.
[42] Wang H, Sun L, Su L, et al. Mixed lineage kinase domain-like protein MLKL causes necrotic membrane disruption upon phosphorylation by RIP3. Mol Cell 2014;54:133–146.
[43] Soffientini U, Beaton N, Baweja S, et al. The lipopolysaccharide-sensing caspase(s)-4/11 are activated in cirrhosis and are causally associated with progression to multi-organ injury. Front Cell Dev Biol 2021;9:668459.
[44] Khanova E, Wu R, Wang W, et al. Pyroptosis by caspase11/4-gasdermin-D pathway in alcoholic hepatitis in mice and patients. Hepatology 2018;67:1737–1753.
[45] Verma N, Garg P, Kaur P, et al. Identification and validation of RIPK3 as a novel biomarker to predict outcomes in patients with acute decompensation of cirrhosis. J Hepatol 2026;84:99–112.
[46] Zhao Q, Chen DP, Chen HD, et al. NK-cell-elicited gasdermin-D-dependent hepatocyte pyroptosis induces neutrophil extracellular traps that facilitate HBV-related acute-on-chronic liver failure. Hepatology 2025;81:917–931.

Keywords: RIPK3; Acute liver failure; Multiorgan failure; Prognosis.
Received 19 January 2026; received in revised form 3 July 2026; accepted 14 July 2026; Available online 4 August 2026

Journal of Hepatology, Volume *8*

Supplemental information

Serum receptor-interacting protein kinase 3 (RIPK3) is associated with transplant-free survival in acute liver failure

Gautam Mehta, Chengyi Ding, Ugo Soffientini, Jaime L. Speiser, Sarah Bahnassi, Lily Dara, Valerie Durkalski, William M. Lee, Constantine J. Karvellas, and US Acute Liver Failure Study Group

Serum receptor-interacting protein kinase 3 (RIPK3) is associated with transplant-free survival in acute liver failure

Gautam Mehta, Chengyi Ding, Ugo Soffientini, Jaime L. Speiser, Sarah Bahnassi, Lily Dara, Valerie Durkalski, William M. Lee, Constantine J. Karvellas, for the US Acute Liver Failure Study Group

Table of contents

Supplementary Figures ……………………………………………………………………3

Supplementary Tables……………………………………………………….……………5

Fig. S1: Patient selection for the nested case-control analysis

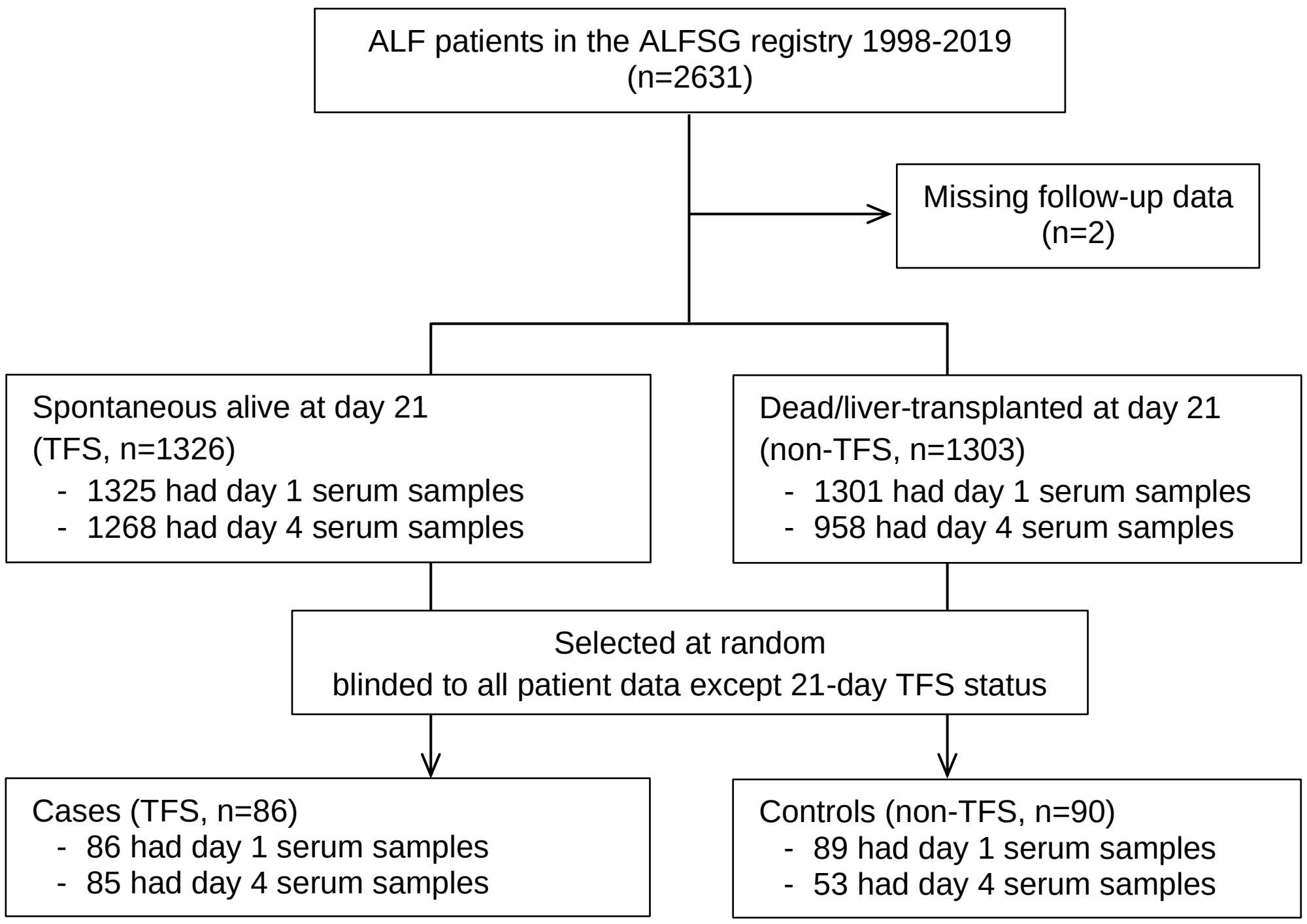

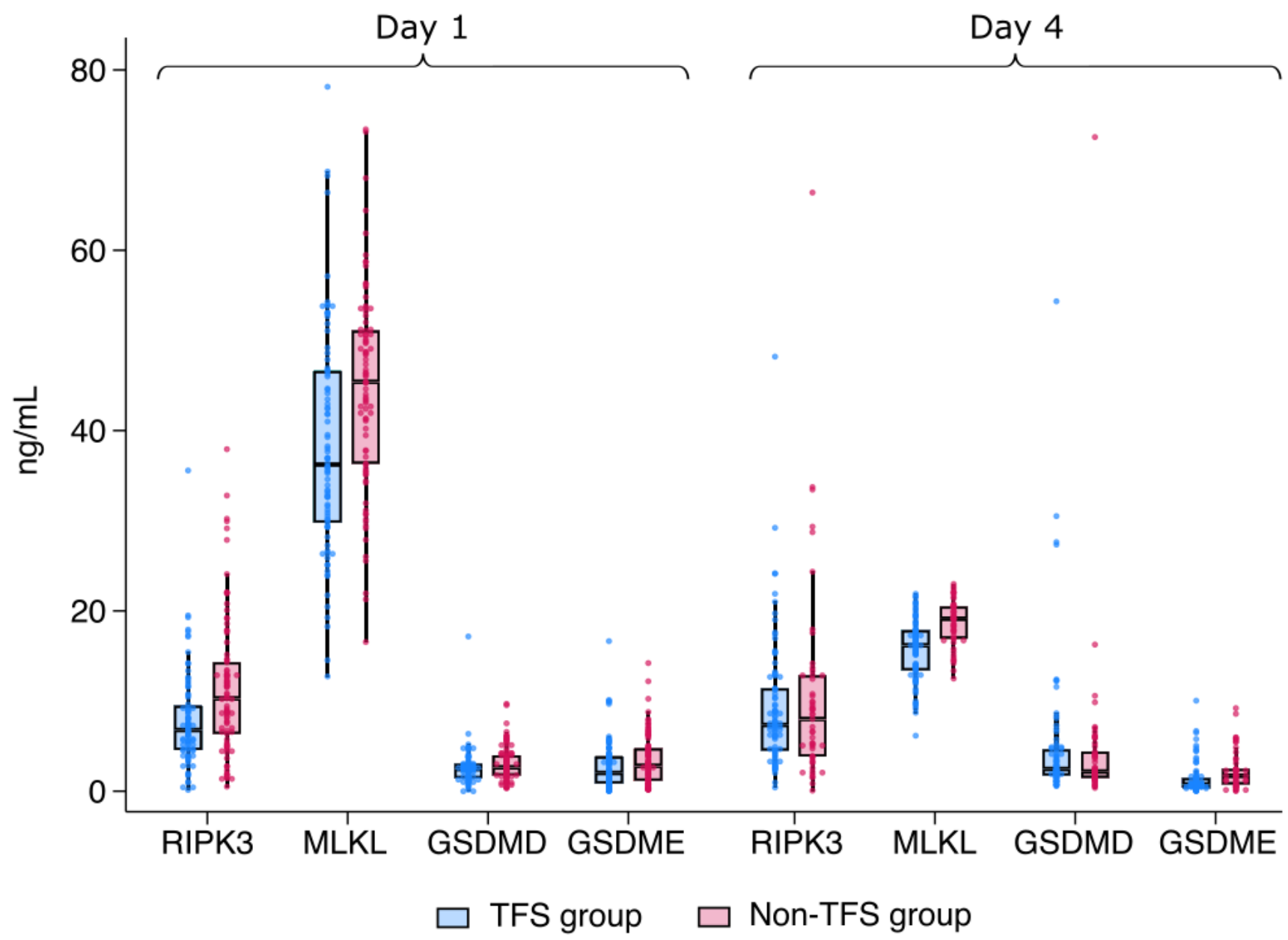

Fig. S2: Day 1 and day 4 levels of RIPK3, MLKL, GSDMD and GSDME in 176 ALF patients

Table S1: RIPK3, MLKL, GSDMD, and GSDME levels (xULN) by 21-day TFS status

	Spontaneous alive at day 21 (TFS, n=86)		Dead/LT at or before day 21 (non-TFS, n=90)		p value
	N	n (%)/median (IQR)	N	n (%)/median (IQR)	
**Day 1**					
RIPK3 xULN	84	2.46 (1.66, 3.43)	89	3.73 (2.31, 5.18)	<0.001
MLKL xULN	86	10.14 (8.34, 13.04)	89	12.71 (10.17, 14.29)	<0.001
GSDMD xULN	86	1.01 (0.60, 1.24)	89	1.08 (0.72, 1.62)	0.074
GSDME xULN	86	0.18 (0.08, 0.34)	89	0.25 (0.10, 0.42)	0.135
**Day 4**					
RIPK3 xULN	85	2.64 (1.61, 4.11)	53	2.90 (1.39, 4.66)	0.947
MLKL xULN	85	4.51 (3.74, 4.97)	53	5.35 (4.73, 5.73)	<0.001
GSDMD xULN	85	0.98 (0.69, 1.87)	53	0.89 (0.61, 1.79)	0.676
GSDME xULN	84	0.06 (0.03, 0.12)	53	0.15 (0.07, 0.22)	<0.001

Normalised as multiples of the ULN (upper limit of normal), based on the maximum value observed in healthy controls (Mann-u-Whitney).

Table S2: Demographics and serum levels of RIPK3, MLKL, GSDMD, and GSDME in healthy controls

	Healthy controls (n=8)
Age (years)	44.5 (44-52)
Male	3 (37.5)
White/Caucasian	8 (100)
RIPK3 (ng/mL)	2.01 (1.57-2.18)
MLKL (ng/mL)	2.86 (2.33-3.26)
GSDMD (ng/mL)	1.65 (1.38-1.87)
GSDME (ng/mL)	2.33 (0.13-6.12)

Data are median (interquartile range) or n (%).

Table S3: RIPK3, MLKL, GSDMD, and GSDME levels (xULN) in association with 21-day TFS

	TFS	N	Adjusted OR (95%CI) *	p value
**Day 1**				
RIPK3 xULN	82	168	1.30 (1.09-1.54)	0.004
MLKL xULN	84	170	1.04 (0.92-1.19)	0.509
GSDMD xULN	84	170	1.52 (0.96-2.43)	0.077
GSDME xULN	84	170	0.89 (0.17-4.67)	0.895
**Day 4**				
RIPK3 xULN	77	123	0.98 (0.81-1.17)	0.787
MLKL xULN	77	123	1.61 (0.77-3.37)	0.202
GSDMD xULN	77	123	0.96 (0.82-1.13)	0.633
GSDME xULN	77	123	1.02 (0.24-4.30)	0.983

* Adjusted for aetiology (APAP or DILI), MELD, vasopressors use, need for renal replacement therapy and mechanical ventilation, all recorded on the same day as the biomarker measurement (day 1 or day 4); per 1x ULN (upper limit of normal) decrease. (Univariate logistic regression)

Table S4: Association of RIPK3, MLKL, GSDMD, and GSDME levels with 21-day TFS, estimated using 1,000 bootstrap iterations

	Adjusted OR (95%CI) *	p value
**Day 1**		
RIPK3	1.10 (1.02-1.19)	0.018
MLKL	1.01 (0.97-1.06)	0.570
GSDMD	1.19 (0.96-1.47)	0.110
GSDME	0.99 (0.85-1.15)	0.899
**Day 4**		
RIPK3	0.99 (0.91-1.08)	0.827
MLKL	1.14 (0.91-1.44)	0.258
GSDMD	0.98 (0.87-1.12)	0.808
GSDME	1.00 (0.78-1.28)	0.991

* Adjusted for aetiology (APAP or DILI), MELD, vasopressors use, need for renal replacement therapy and mechanical ventilation, all recorded on the same day as the biomarker measurement (day 1 or day 4); per 1 ng/mL decrease. (logistic regression)

Table S5: Contributions of admission clinical variables to FAMD dimensions

Variable	Dim 1 (%)	Dim 2 (%)	Dim 3 (%)	Dim 4 (%)	Dim 5 (%)
Age (years)	0.05	1.48	4.47	1.42	0.14
Hemoglobin (g/dL)	6.67	15.81	5.85	7.79	2.96
White Blood count (10^9/L)	1.20	1.34	10.33	0.05	23.88
Lymphocytes (10^9/L)	3.32	4.13	0.33	0.91	5.19
Platelet count (10^9/L)	2.69	1.44	1.39	4.34	22.95
ALT (IU/L)	2.24	20.94	5.19	1.22	0.70
AST (IU/L)	5.89	16.32	2.05	3.60	0.02
Bilirubin (mg/dL)	3.03	5.05	15.38	0.37	0.21
Creatinine (mg/dL)	8.35	0.01	5.51	4.58	4.69
Phosphate (mg/dL)	5.57	0.02	21.94	4.65	0.29
PH	5.82	1.58	5.79	7.44	1.87
Hematocrit	6.23	16.89	6.03	7.00	1.90
INR	0.51	7.10	0.18	1.62	2.33
HE coma grade	9.20	1.11	0.05	25.98	0.03
Male	0.08	1.17	7.52	0.35	27.29
Mechanical ventilation	12.42	0.71	0.27	22.17	0.01
Vasopressors use	12.86	0.06	0.98	3.60	0.03
Race	0.33	2.71	1.46	1.68	3.38
Aetiology (APAP/DILI)	1.46	1.54	4.53	0.16	0.22
Renal replacement therapy	12.06	0.59	0.75	1.08	1.93

Table S6: Admission characteristics of FAMD-based k-means clusters in ALFSG ALF patients

	Cluster 1 (n=1599)	Cluster 2 (n=1020)	p value
Age (years)	41 (30-53)	41 (30-53)	0.595
HE coma grade	2 (1-3)	4 (3-4)	<0.001
Biochemistry			
Hemoglobin (g/dL)	11.6 (10.2-13.2)	9.8 (8.7-11.3)	<0.001
White Blood count (10^9/L)	9.5 (6.8-13.7)	11.4 (7.2-17.1)	<0.001
Lymphocytes (10^9/L)	10.8 (5.9-18.0)	7.0 (4.0-11.0)	<0.001
Monocytes (10^9/L)	6.0 (2.5-10.0)	3.0 (1.0-6.0)	<0.001
Platelet count (10^9/L)	144 (96-203)	102 (64-153)	<0.001
INR	2.7 (2.0-4.0)	2.8 (2.0-4.4)	0.016
ALT (IU/L)	1602 (500.5-3786.5)	2640.5 (947-5045)	<0.001
AST (IU/L)	1147.5 (390-3283)	2752 (721.5-7164.5)	<0.001
Bilirubin (mg/dL)	8.9 (3.9-22.1)	5.5 (3.4-10.5)	<0.001
Ammonia (venous) (µmol/L)	88 (57-133)	115 (73-178)	<0.001
Creatinine (mg/dL)	1.1 (0.7-2.0)	2.7 (1.7-4.2)	<0.001
Lactate (mmol/L)	2.9 (1.9-5.0)	5.8 (3.3-10.5)	0.461
Phosphate (mg/dL)	2.7 (1.8-3.7)	3.7 (2.6-5.6)	<0.001
Aetiology			
APAP	1378 (86.2)	961 (94.2)	<0.001
DILI	221 (13.8)	59 (5.8)	
Male	520 (32.5)	305 (29.9)	0.173
Race			
White/Caucasian	1190 (74.4)	782 (76.7)	0.037
African American	254 (15.9)	132 (12.9)	
Other	155 (9.7)	106 (10.4)	
Organ support			
Mechanical ventilation	358 (22.4)	870 (85.3)	<0.001
Vasopressors use	43 (2.7)	509 (49.9)	<0.001
Renal replacement therapy	53 (3.3)	545 (53.4)	<0.001

Data are median (interquartile range) or n (% of available data). (Mann-U-Whitney for continuous variables, chi-squared for categorical)

Table S7: K-means cluster-specific associations of RIPK3, MLKL, GSDMD, and GSDME levels on day 1 with 21-day TFS

	Cluster 1 (TFS=54/n=86)		Cluster 2 (TFS=32/n=90)	
	Adjusted OR (95%CI) *	p value	Adjusted OR (95%CI) *	p value
**Per 1 ng/mL decrease**				
RIPK3	1.04 (0.96-1.12)	0.322	1.13 (1.03-1.27)	0.017
MLKL	1.02 (0.97-1.08)	0.404	1.01 (0.96-1.06)	0.805
GSDMD	1.12 (0.89-1.41)	0.314	1.38 (0.99-2.10)	0.088
GSDME	0.89 (0.72-1.06)	0.220	1.15 (0.91-1.50)	0.253
**Per 1x ULN decrease**				
RIPK3 xULN	1.11 (0.90-1.37)	0.322	1.40 (1.09-1.92)	0.017
MLKL xULN	1.08 (0.90-1.30)	0.404	1.02 (0.86-1.22)	0.805
GSDMD xULN	1.31 (0.75-2.33)	0.314	2.21 (0.97-6.14)	0.088
GSDME xULN	0.26 (0.02-1.95)	0.220	5.07 (0.36-100.62)	0.253

* Adjusted for aetiology (APAP or DILI), MELD, vasopressors use, need for renal replacement therapy and mechanical ventilation, all recorded on day 1.

**Univariable and multivariable logistic regression, Wald test.

Table S8: Admission characteristics of FAMD-based GMM clusters in ALFSG ALF patients

	Cluster 1 (n=1488)	Cluster 2 (n=1131)	p value
Age (years)	42 (30-55)	39 (29-51)	<0.001
HE coma grade	2 (1-2)	4 (3-4)	<0.001
Biochemistry			
Hemoglobin (g/dL)	11.5 (9.9-13.1)	10.2 (9.0-11.8)	<0.001
White Blood count (10^9/L)	9.8 (6.9-14.3)	10.7 (6.8-16.0)	0.011
Lymphocytes (10^9/L)	10.0 (5.0-17.0)	7.9 (4.8-14.0)	0.001
Monocytes (10^9/L)	5.5 (2.0-10.0)	4.0 (1.0-7.1)	<0.001
Platelet count (10^9/L)	134 (89-193)	115 (71-175)	<0.001
INR	2.70 (2.00-3.88)	2.80 (2.00-4.47)	0.001
ALT (IU/L)	1,705 (479.8-4,175.2)	2,310.5 (834-4,443)	0.201
AST (IU/L)	1,406.0 (411.0-4,326.0)	2,106 (582.0-5,718.0)	<0.001
Bilirubin (mg/dL)	8.0 (3.6-21.8)	6.3 (3.6-13.2)	<0.001
Ammonia (venous) (μmol/L)	83 (56-127)	116 (77-177)	<0.001
Creatinine (mg/dL)	1.3 (0.8-2.6)	2.1 (1.1-3.5)	<0.001
Lactate (mmol/L)	3.0 (1.9-5.6)	5.0 (3.0-9.6)	0.218
Phosphate (mg/dL)	3.0 (2.1-4.3)	3.1 (2.1-4.7)	0.010
Aetiology			
APAP	1,278 (85.9)	1,061 (93.8)	<0.001
DILI	210 (14.1)	70 (6.2)	<0.001
Male	488 (32.8)	337 (29.8)	0.111
Race			
White/Caucasian	1,126 (75.7)	846 (74.8)	0.022
African American	229 (15.4)	157 (13.9)	<0.001
Other	133 (8.9)	128 (11.3)	<0.001
Organ support			
Mechanical ventilation	127 (8.5)	1,101 (97.3)	<0.001
Vasopressors use	65 (4.4)	487 (43.1)	<0.001
Renal replacement therapy	116 (7.8)	482 (42.6)	<0.001

Data are median (interquartile range) or n (% of available data).
(Mann-U-Whitney for continuous variables, chi-squared for categorical)

Table S9: GMM cluster-specific associations of RIPK3, MLKL, GSDMD, and GSDME levels on day 1 with 21-day TFS

	Cluster 1 (TFS=54/n=83)		Cluster 2 (TFS=32/n=93)	
	Adjusted OR (95%CI) *	p value	Adjusted OR (95%CI) *	p value
**Per 1 ng/mL decrease**				
RIPK3	1.03 (0.95-1.11)	0.500	1.14 (1.04-1.27)	0.014
MLKL	1.01 (0.96-1.06)	0.739	1.02 (0.97-1.08)	0.488
GSDMD	1.09 (0.85-1.37)	0.448	1.53 (1.05-2.53)	0.054
GSDME	0.92 (0.75-1.10)	0.393	1.08 (0.87-1.40)	0.501
**Per 1x ULN decrease**				
RIPK3 xULN	1.08 (0.86-1.34)	0.500	1.42 (1.10-1.95)	0.014
MLKL xULN	1.03 (0.86-1.23)	0.739	1.07 (0.89-1.30)	0.488
GSDMD xULN	1.23 (0.67-2.17)	0.448	2.83 (1.14-9.68)	0.054
GSDME xULN	0.39 (0.04-2.92)	0.393	2.49 (0.19-44.93)	0.501

* Adjusted for aetiology (APAP or DILI), MELD, vasopressors use, need for renal replacement therapy and mechanical ventilation, all recorded on day 1.

**Univariable and multivariable logistic regression, Wald test.

Table S10: Performance of models in predicting 21-day TFS

	TFS	N	AUROC (95%CI)
**Day 1**			
KCC	86	176	0.57 (0.51, 0.63)
KCC+RIPK3	84	173	0.67 (0.59, 0.75)
KCC+MLKL	86	175	0.70 (0.62, 0.78)
MELD	84	171	0.72 (0.64, 0.80)
MELD+RIPK3	82	168	0.75 (0.68, 0.83)
MELD+MLKL	84	170	0.73 (0.65, 0.80)
ALFSG Index	81	166	0.80 (0.73, 0.87)
ALFSG Index+RIPK3	79	163	0.83 (0.76, 0.89)
ALFSG Index+MLKL	81	165	0.82 (0.75, 0.88)
RIPK3+MELD+Vasopressors use+MV	82	168	0.87 (0.81, 0.92)
**Day 4**			
KCC	86	176	0.57 (0.51, 0.63)
KCC+MLKL	85	138	0.77 (0.69, 0.85)
KCC+GSDME	84	137	0.70 (0.61, 0.79)
MELD	78	134	0.82 (0.74, 0.89)
MELD+MLKL	77	123	0.83 (0.76, 0.91)
MELD+GSDME	76	122	0.81 (0.74, 0.89)
ALFSG Index	60	116	0.84 (0.77, 0.91)
ALFSG Index+MLKL	59	105	0.86 (0.79, 0.93)
ALFSG Index+GSDME	58	104	0.85 (0.78, 0.93)

ALFSG= Acute Liver Failure Study Group, AUROC=area under the ROC curve, KCC= King's College Criteria, MELD= Model for End-stage Liver Disease, MV= mechanical ventilation, TFS=transplant-free survival.

Table S11: Cross-validated predictive performance of models

	mean AUROC (95%CI)	Paired t-test p value		
		vs. KCC	vs. MELD	vs. ALFSG Index
**Day 1**				
KCC	0.57 (0.35, 0.53)	NA		
KCC+RIPK3	0.67 (0.56, 0.73)	<0.001		
KCC+MLKL	0.71 (0.57, 0.75)	0.021		
MELD	0.73 (0.62, 0.78)		NA	
MELD+RIPK3	0.75 (0.65, 0.81)		0.624	
MELD+MLKL	0.74 (0.61, 0.77)		0.884	
ALFSG Index	0.81 (0.70, 0.85)			NA
ALFSG Index+RIPK3	0.81 (0.71, 0.86)			0.959
ALFSG Index+MLKL	0.80 (0.74, 0.88)			0.820
RIPK3+MELD+Vasopressors use+MV	0.85 (0.77, 0.89)			<0.001
**Day 4**				
KCC	0.57 (0.35, 0.53)	NA		
KCC+MLKL	0.76 (0.66, 0.83)	<0.001		
KCC+GSDME	0.70 (0.58, 0.77)	0.008		
MELD	0.83 (0.71, 0.87)		NA	
MELD+MLKL	0.83 (0.72, 0.89)		0.879	
MELD+GSDME	0.81 (0.73, 0.89)		0.516	
ALFSG Index	0.81 (0.73, 0.89)			NA
ALFSG Index+MLKL	0.87 (0.75, 0.92)			0.221
ALFSG Index+GSDME	0.85 (0.78, 0.93)			0.426

ALFSG= Acute Liver Failure Study Group, AUROC=area under the ROC curve, KCC= King's College Criteria, MELD= Model for End-stage Liver Disease, MV= mechanical ventilation, TFS=transplant-free survival.

Table S12: Temporal changes in RIPK3, MLKL, GSDMD, and GSDME in association with 21-day TFS

	TFS	N	OR (95%CI) *	p value
**Adjusted for day 1 level**				
ΔRIPK3 (Δ=day 4-day 1, ng/mL)	83	135	0.99 (0.95, 1.04)	0.725
ΔMLKL	85	137	1.47 (1.23, 1.75)	<0.001
ΔGSDMD	85	137	1.00 (0.96, 1.04)	0.828
ΔGSDME	84	136	1.30 (1.07, 1.59)	0.010
**Adjusted for day 1 level + clinical covariates** **				
ΔRIPK3	81	131	0.99 (0.94, 1.04)	0.559
ΔMLKL	83	133	1.24 (1.02, 1.52)	0.035
ΔGSDMD	83	133	0.99 (0.94, 1.05)	0.859
ΔGSDME	82	132	1.26 (0.99, 1.62)	0.063

* Per 1 ng/mL larger decrease.

** Etiology (APAP or DILI), MELD, vasopressors use, need for renal replacement therapy and mechanical ventilation, all recorded on day 1.

***Logistic regression, Wald test.
